# Aqueous hybrid electrochemical capacitors with ultra-high energy density approaching for thousand-volts alternating current line filtering

**DOI:** 10.1038/s41467-022-34082-2

**Published:** 2022-10-26

**Authors:** Zhou Li, Xiaopeng Wang, Lingyu Zhao, Fengyao Chi, Chang Gao, Ying Wang, Mengdan Yan, Qian Zhou, Miaomiao Zhao, Xinyang Wang, Jiaqi Wang, Man Yuan, Mingmao Wu, Lixia Wang, Yang Zhao, Liangti Qu

**Affiliations:** 1 College of Science, Henan Agricultural University, Zhengzhou, Henan 450002 China; 2 Key Laboratory of Cluster Science, Ministry of Education of China, School of Chemistry and Chemical Engineering, Beijing Institute of Technology, Beijing, 100081 China; 3 Department of Chemistry, Tsinghua University, 100084 Beijing, P. R. China; 4 Key Laboratory of Eco-materials Advanced Technology, College of Materials Science and Engineering, Fuzhou University, Fuzhou, 350108 P. R. China

**Keywords:** Supercapacitors, Porous materials, Electrical and electronic engineering

## Abstract

Filtering capacitors with wide operating voltage range are essential for smoothing ripples in line-powered system, which are still unsatisfactory due to low energy density and limited working voltage scopes. Herein, we report an aqueous hybrid electrochemical capacitor with areal specific energy density of 1.29 mF V^2^ cm^−2^ at 120 Hz, greater than common aqueous ones. Interestingly, it can be easily integrated at scale to show excellent flexibility, controllable and stable filtering performance, in which an integrated device (e.g., seven units in series) exhibits fluctuation of 96 mV, 10 times smaller than an aluminum electrolytic capacitor with similar capacitance. A record-high 1,000 V can also be achieved after integrating 670 units, exceeding those reported so far, and about 1.5 times of commercial bulk aluminum electrolytic capacitors (~700 V). This work opens up a new insight for promising applications in multiple electricity transmission systems that requiring high smoothness under harsh voltage.

## Abstract (web summary)

Filtering capacitors are essential to smooth high voltage alternating current lines but are typically limited to hundreds of volts. Here, the authors demonstrate an aqueous hybrid electrochemical capacitor that can exhibit an operating voltage of 1,000 V when assembled into a device of 670 units.

## Introduction

Alternating current to direct current (AC-DC) conversion element with shape-configuration, wide frequency and voltage ranges has become increasingly indispensable part for stabilizing and smoothing signal ripples in next-generation electric power systems^1–4^, especially in the promising sustainable electronic devices capable of generating high voltage. As typical filtering capacitors, aluminum electrolytic capacitors (AECs) can be generally used for AC line filtering in the high operating voltage ranges. However, except for bulky and rigid appearances that are unable to match the electronic integrated system, the maximum voltage of AECs is usually restricted to hundreds of volts due to the inherent low withstand voltage of the alumina dielectric layer, far from satisfactory in the high voltage AC-DC system^5–8^.

Electrochemical capacitors (ECs) with the advantages of adjustable and compact configuration, higher areal-specific energy density and long cycle life, are attractive candidates to replace AECs for multi-application scenarios^9–12^. Although various electrode materials, including carbon-based materials^9,10,13–29^, conductive polymers^4,30–32^, Prussian blue^33^ and MXene^34,35^, have been employed into fabricating ECs for filtering, their low capacitance, large equivalent series resistance (ESR), complicated preparation process and inconsistent connection hinder the development of the high-voltage integration of ECs. It is desirable to obtain ECs with excellent filtering performance in broad voltage and frequency ranges, which not only relies on the inherent electrochemical capacity of electrodes, but also requires the high compatibility and structural stability of capacitor units during integration.

Apart from the structural control over the electrode materials, the performance of filtering capacitors can also be further improved by expanding the working voltage window. Recent achievements have shown that the non-aqueous and water-in-salt electrolytes could effectively increase the applicable voltage range (e.g., ~3 V), but at the expense of poor ionic conductivity, environmentally unfriendly, high cost, strong corrosivity, danger and troublesome operation process^19,28,33,36^, which will lead to severe attenuation of the filtering performance during high-voltage integration. In contrast, the common aqueous electrolyte-based ECs with fast frequency response can avoid above shortcomings, and achieve a higher filtering working voltage through rational integration of capacitor units^2,30,37–39^. In this regard, we have verified that a maximum operating voltage of ~200 V could be obtained after proper integration in common aqueous environment^30^. Unfortunately, the low areal specific capacitance (*C*_A, 120_ < ~ 0.3 mF cm^−2^) at 120 Hz of the single capacitor unit will make it difficult to achieve a desired high voltage (e.g., thousand-volts) for filtering in a limited volume size through integration. Therefore, there is still great need to explore and develop ECs with excellent areal-specific energy density (including high areal-specific capacitance and wide voltage window) and fast frequency response, as well as the exquisite integration technics with flexible configuration and intact packaging, to achieve AC line filtering with high performance for wide voltage range applicable possibilities.

Herein, we report an aqueous hybrid electrochemical capacitor with continuous PEDOT nanomesh film (CPN film) as the positive electrode and porous carbon nanotube film (p-CNT film) as the negative electrode (abbreviated as ACPEC). With the virtue of fast charge transport facilitated by the highly conductive and interpenetrating networks of the electrodes materials, the ACPEC exhibits excellent rate performance with a phase angle of –83.3° and a small resistance-capacitance time constant (*τ*_RC_) of 0.15 ms. It also exhibits areal specific energy density (*E*_A, 120_) of 1.29 mF V^2^ cm^−2^ (0.36 μW h cm^−2^) at 120 Hz, which is higher than that of common aqueous filtering capacitors reported previously. We also find that the flexible and mechanically stable electrode films make the reprocessing and integration of the capacitor units possible. Various integrated ACPEC devices, such as 7 units, 67 units and even 670 units connected in series, are successfully achieved by applying a scalable orderly aligned scrolling (OAS) strategy. Interestingly, the integrated device with 7 units (7-ACPECs, ~10 V, 40 μF) presents excellent filtering performance with an ultra-low fluctuation of 96 mV, which is 10 times smaller than an AEC (47 μF/ 16 V, CHONGX, China) of 1 V. After bending in different states, it retains steady phase angle and capacitance (*C*_120_) at 120 Hz, indicating the excellent flexibility of the integrated device. Moreover, the device containing 67 units (67-ACPECs) can be easily integrated into the filtering circuit, in which the output pulsed AC signal from a rotating disk triboelectric nanogenerator (RD-TENG) can be converted into a stable and continuous DC signal of 100 V, promoting the TENG as a power source for practical use. Notably, a flexible device consisting of 670 units (670-ACPECs) can be further integrated by scrolling into a scroller-like shape with a radius of 4 cm (smaller than a commercial disk). It exhibits an ultrahigh voltage of 1000 V, approximately unchanged fast frequency response performance with a phase angle of –76° and a resistance-capacitance time (*τ*_RC_) of 0.33 ms. The energy of 670-ACPECs (*E*_120_) reaches 250 mF V^2^ (69.5 μW h) at 120 Hz, suggesting the high compatibility of the assembled components in the filtering system. The reasonable and scalable integration method demonstrated in this work would provide more possibilities for the practical application of filtering capacitors in multifarious power transmission systems.

## Results and discussion

### Fabrication and characterization of ACPEC

**Fig. 1:**
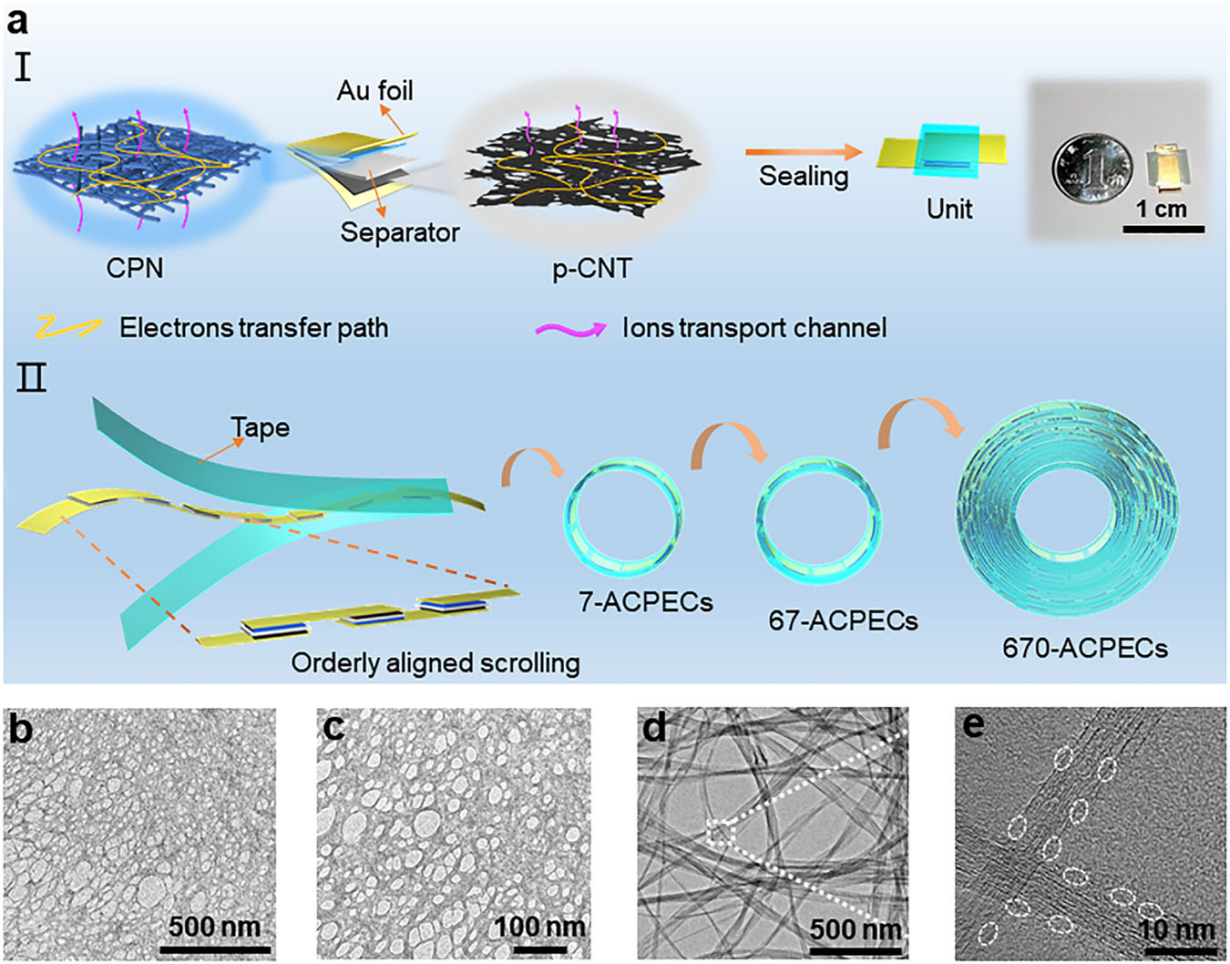
The preparation strategy of filtering capacitors with wide operating voltage ranges and the characterization of electrode materials. **a** Schematic of fabrication processes of a single ACPEC unit (I); and the integrated filtering capacitors consist of different ACPEC units connected in series (II). **b, c** TEM images of the positive CPN film electrode material and **d, e** negative p-CNT film electrode material under different magnifications, respectively. The white dotted circles in (**e**) indicate pore defects over the CNT walls of p-CNT film.

The single ACPEC unit was assembled with a sandwich configuration (Fig. 1a (I)). To realize highly efficient AC line filtering, the fast frequency responsiveness and large areal-specific capacitance of ACPEC are necessary, which usually require the electrodes featured with superior conductivity, fast charge transport process, and electrochemical activity. Here, using porous cellulose paper as a template^4^, a continuous PEDOT nanomesh (CPN) film-based positive electrode was synthetized by spinning the PEDOT:PSS/DMSO mixed solution on cellulose paper, which was then treated with concentrated sulfuric acid and rinsed with deionized water (the conductivity of 2.7 × 10^3^ S cm^−1^, Fig. 1b, c, Supplementary Fig. 1, 2 and Supplementary Movie 1). While, a porous carbon nanotube (p-CNT) film exfoliated from CNT paper after etching with H_2_O_2_ was used as negative electrode (the conductivity of 1.8 × 10^3^ S cm^−1^, Fig. 1d, Supplementary Fig. 1, 2 and Supplementary Movie 2), which presents rough and porous tube walls (Fig. 1e and Supplementary Fig. 3). The Brunauer-Emmett-Teller (BET) test further revealed the presence of the pore structures in p-CNT film with two main size distributions of below ~6 nm and 10 ~60 nm (Supplementary Fig. 4), which may come from pore defects on the CNT walls and loosely interconnected network of p-CNT film. Both of CPN film and p-CNT film show superior flexibility, highly conductive and interpenetrating framework, which provide fast charge transport and abundant exposed area for ions adsorption/desorption (Supplementary Figs. 5, 6). After assembling the two electrodes on Au foils (as the current collectors), the compact ACPEC unit (0.7 × 0.9 × 0.07 cm^3^) was finally obtained by using cellulose diaphragm (thickness of 40 μm) moistened with 3 M sulfuric acid as the separator and sealed by parafilm, which is approximately 2 times smaller than a Chinese dime coin (Fig. 1a (I), 3.14 × 0.5^2^ × 0.2 cm^3^). Interestingly, the ACPEC units can be easily integrated in a large scale through the OAS assembly strategy. As illustrated in Fig. 1a (II), the ACPEC units can be sequentially assembled into strip-shaped integrated devices. After sealing with soft tapes (high viscosity thermal release tape and biaxially oriented polypropylene tape (BOPP)), the strip-shaped capacitors can be wound into a bobbin to achieve the integrated filtering devices with more units, which are named as n-ACPECs (e.g., 7-ACPECs, 67-ACPECs, and 670-ACPECs). The fabrication details of electrode materials (Supplementary Fig. 7) and integrating process of ACPEC units (Supplementary Fig. 8) can be found in Methods’ Section.

In order to further investigate the structure and components of the positive and negative electrode materials, Raman spectroscopy and X-ray photoelectron spectroscopy (XPS) isotherms were performed. For the CPN film, the conductivity is greatly improved to 2.7 × 10^3^ S cm^−1^ in comparison with 10 S cm^−1^ of pristine PEDOT:PSS film. The conductive enhancement could be attributed to the reduced ratio of PSS and increased conjugation length of PEDOT chain in the film, which can be proven in XPS (Supplementary Fig. 9a) and Raman (Supplementary Fig. 9b) results. For the p-CNT film, in Raman spectroscopy, the *I*_D_/*I*_G_ value of 0.54 for p-CNT film is larger than that of pristine CNTs paper (*I*_D_/*I*_G_ = 0) (Supplementary Fig. 10)^40,41^, indicating the existence of defects in the graphitic layer of the p-CNT film after H_2_O_2_ etching. This is consistent with the TEM observations and BET results. Similar result can also be found in the XPS data (Supplementary Fig. 11). The oxygen content of p-CNT film slightly increases after treatment, suggesting the introduction of oxygen-containing groups after H_2_O_2_ etching process. As a result of this, the p-CNT film exhibits a decreased conductivity (1.8 × 10^3^ S cm^−1^) than that of the pristine CNT paper (2.8 × 10^3^ S cm^−1^). Interestingly, we find that the formation of pore structures plays an important role in the promotion of the electrochemical process. To investigate the electrochemical behaviors of pore structures in p-CNT film, the CNT film with similar thickness directly exfoliated from CNT paper without H_2_O_2_ etching (named as CNT film) is used as a comparison (Supplementary Figs. 12, 13). Compared with CNT film, the electrolyte permeability of the p-CNT film is effectively improved through the formation of pores and oxygen-related groups, which can be reflected from the enhancement of hydrophilicity for the p-CNT film electrode (Supplementary Fig. 13a, b). A p-CNT film-based symmetrical supercapacitor (p-CNTEC) presents a smaller equivalent series resistance (ESR) of 0.32 Ω than that of CNT film-based one (CNTEC, 0.68 Ω, supplementary Fig. 13c), indicating the small interfacial resistance between p-CNT film and current collector. Meanwhile, the Nyquist plot of p-CNTEC shows the absence of 45° region in high-frequency region, indicating porous electrode behavior is not obvious. It demonstrates that surface charge mainly participates in the contribution to the electric double layer under high frequency, that is, the effective electrochemical surface area may be mainly from external defects and surface of tube walls. This can also be reflected in a higher areal capacitance (1.03 mF cm^−2^) of the p-CNTEC than that of CNTEC (0.81 mF cm^−2^, supplementary Fig. 13d) at 120 Hz, indicating the high electrochemical surface area of p-CNT film. From the above analysis, we speculate that pore defects over p-CNT walls could contribute to high infiltration capability of electrolyte and effective ion-accessible active surface, thus facilitating the electrochemical process^2^.

### Electrochemical performance of ACPEC unit

**Fig. 2:**
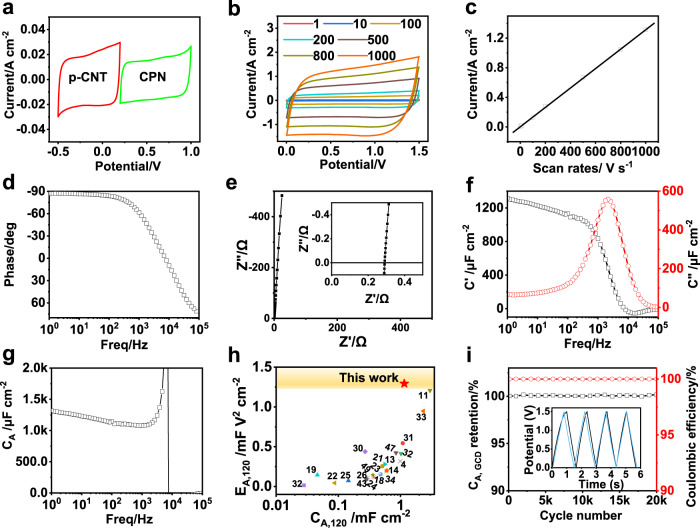
The electrochemical performance of electrodes and unit. **a** The CV curves of the positive CPN film electrode and negative p-CNT film electrode at the scan rate of 10 V s^−1^. **b** The CV curves of ACPEC at different scan rates from 1~1000 V s^−1^. **c** Scan rates versus discharging current density of ACPEC. **d**–**g** The electrochemical impedance spectroscopy (EIS) test of an ACPEC unit. d) Bode plot. **e** Nyquist plot, inset: the expanded view at high frequencies. **f** Plots of the real or imaginary parts of specific capacitance (*C*’ or *C*”) versus frequency. **g** Plot of areal-specific capacitance (*C*_A_) as a function of frequency. **h** Comparison of areal specific capacitance (*C*_A, 120_) and areal specific energy density (*E*_A, 120_) of the ACPEC at 120 Hz with those of the reported common aqueous AC line filtering ECs (Supplementary Table S1)^4,11,13,14,18,19,21–26,30–34,43,47,49^. i) Cycling stability of the ACPEC calculated from GCD curves at a discharging at a current density of 5 mA cm^−2^ for 20,000 cycles, inset view: the GCD curves of the first and last 4 cycles. Source data are provided as a Source Data file.

To determine the appropriate voltage window, the electrochemical performances of positive and negative electrodes were studied with linear scan voltammetry (LSV), cyclic voltammetry (CV) and galvanostatic charge-discharge (GCD) tests. From the results of LSV test in three-electrode system, the operating voltage windows of the positive and negative electrodes were selected as 0.2~1 V and –0.5~0.2 V *vs*. Ag*@*AgCl, respectively (Supplementary Fig. 14). The hybrid ACPEC device was then assembled according to the principle of charge balance, and the loads of the positive and negative active materials were adjusted to prevent the electrodes from overcharging (Fig. 2a, Eqs. 1 and 2, here the thickness of each electrode is selected as 200 nm). To further verify the rationality of the selected voltage window, the CV curves of the positive CPN film electrode and negative p-CNT film electrode were tested in three-electrode system within the voltage window of 0.2~1 V and –0.5~0.2 V *vs*. Ag*@*AgCl under different scan rates (1~100 V s^−1^), respectively. All of the CV curves exhibit quasi-rectangular shapes, indicating typical capacitive behaviors (Supplementary Figs. 15a and 16a). After 10,000 cycling tests, the capacitance retentions of positive and negative electrodes can maintain about 92 and 100%, respectively, indicating good electrochemical stabilities of the two electrode materials (Supplementary Figs. 15b and 16b). After assembled into two-electrode device, the rate performance of the ACPEC unit was tested with CV and GCD experiments. As demonstrated in Fig. 2b, the CV curves show stable and similar quasi-rectangular shapes at the scan rates range from 1 to 1000 V s^−1^, indicating the ideal double-layer capacitive behavior and ultrafast charge/discharge capability of the device. The discharge current density shows an ideal linear relationship with the scan rates in the range of 1~1000 V s^−1^ (Fig. 2c), demonstrating fast charge transport within electrode under rapid change of external voltage^4^. These characteristics are also observed from GCD test, in which all of the curves are symmetrical triangles and the capacitance retention of ACPEC unit can still retain at 81% even under the high current density of 50 mA cm^−2^ (2.6 mF cm^−2^) in comparison with that of 1 mA cm^−2^ (3.2 mF cm^−2^) (Supplementary Fig. 17a, b).

The electrochemical performance of the ACPEC unit was further evaluated by electrochemical impedance spectroscopy (EIS). The phase angle reaches to –83.3° at 120 Hz (Fig. 2d), which is comparable to that of commercial AEC (220 μF/16 V, CHONGX, China, Supplementary Fig. 18), and much beyond that of aqueous electrochemical capacitors, such as the PEDOT-based ECs^4,30–32,42^ and carbon-based ECs^9,11,14,17,23,24,28,43–46^. In the Nyquist plot, there is an almost vertical line at low frequencies (Fig. 2e), suggesting a pure capacitive behavior. The absence of semicircle and 45° region in high frequency implies that there is no passive layer between electrodes and the gold current collector next to it, as well as a negligible porous electrode behavior in the electrodes^22,46^. The ESR of 0.07 Ω cm^2^ (the size of electrode is 0.5 × 0.5 cm^2^) for ACPEC unit is smaller than most of filtering capacitors reported so far^4,10,19,21,23,27,47,48^. Except this, the materials on Au foil current collectors remain intact even after stirring in deionized water overnight (Supplementary Fig. 19), indicating the intimate physical contact between the electrode materials and the Au foils. Besides, the resistance-capacitance time constant (*τ*_RC_) is a critical parameter reflecting how fast the capacitor can be charged/discharged. The *τ*_RC_ of ACPEC is 0.15 ms, smaller than that of AEC (0.18 ms, 220 μF/16 V, CHONGX, China) and most of aqueous filtering capacitors (Supplementary Table S1), demonstrating the high-rate performance of ACPEC. The high-frequency response can also be reflected from the dissipation factor at 120 Hz (*DF*_120_), which indicates the degree of energy loss as heat dissipation^25^. The *DF*_120_ of a single ACPEC unit is 10.7% (Supplementary Table S2), lower than that of MXene/PEDOT:PSS hybrid materials (19.2%)^34^, and carbonized Prussian blue cubes with graphene deposition hybrid materials (16.6%)^33^, indicating that little energy of ACPEC unit is dissipated as heat. From the frequency at the maximum *C*” (*f*_0_ = 2153 Hz), the relaxation time constant *τ*_0_ (*τ*_0_ = 1/*f*_*0*_) can be calculated to be 0.46 ms, which also represents a rapid frequency response (Fig. 2f)^46^. Moreover, the areal-specific capacitance measured at 120 Hz (*C*_A, 120_) is 1.15 mF cm^−2^ (Fig. 2g), higher than that of bare Au foil-based EC (Supplementary Fig. 20) or other reported aqueous filtering capacitors (Supplementary Table S1). In addition, the *C*_A, 120_ of ACPEC can also be easily adjusted by increasing the electrode load on the Au foils without obvious sacrificing its high-rate capability (Supplementary Fig. 21).

Combining with the voltage window of 1.5 V, the calculated areal specific energy density of ACPEC at 120 Hz (*E*_A, 120_) is approximately 1.29 mF V^2^ cm^−2^ (0.36 μW h cm^−2^), greater than that of common aqueous filtering capacitors reported till date^4,11,13,14,18,19,21–26,30–34,43,47,49^ (Fig. 2h, Supplementary Table S1). The comparison of ACPEC with water-in-salt filtering capacitor is also included in Supplementary Table S1. The reason for high-rate performance, large areal specific capacitance and energy density of ACPEC is mainly attributed to that the highly conductive backbone and porous network structure of electrode materials provide bicontinuous channels for charge transport, as well as abundant exposed ions accessible area. Furthermore, ACPEC exhibits excellent electrochemical stability (Fig. 2i). Both of the capacitance retention and the coulombic efficiency are approximately 100% after 20,000 cycles of repeated charge/discharge at the current density of 5 mA cm^−2^, which can be clearly observed from the almost coincide GCD curves of the first and last 4 cycles. And they are almost unchanged even after tested for 8.5 × 10^6^ cycles, (Supplementary Fig. 22).

### Filtering performance of a single ACPEC unit

**Fig. 3:**
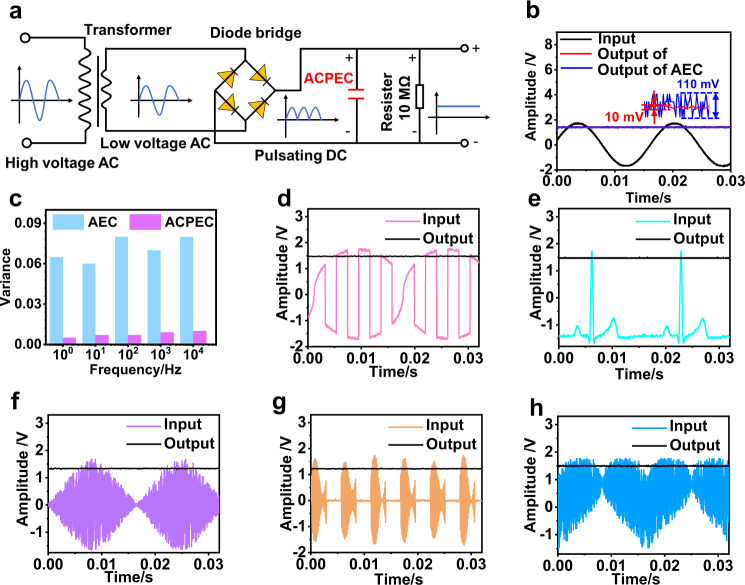
The filtering performance of an ACPEC unit. **a** Schematic demonstration of the filtering circuit. The resistance of load resistor is 10 MΩ. **b** Comparison of AC line filtering performance of the ACPEC with that of the commercial AEC (220 μF/ 16 V, CHONGX, China) at 60 Hz. Inset shows the fluctuations of the DC voltage output signals of ACPEC unit and AEC (220 μF/ 16 V, CHONGX, China). **c** Histogram of variance of the output signals data of ACPEC and AEC versus frequency in the frequency range of 1~10^4^ Hz. **d–h** The filtering performance of the ACPEC unit for arbitrary waveforms. The input alternating current waveform signals were (**d**) rounds PM, (**e**) electrocardiogram, (**f**) diamond, (**g**) Lorentz and (**h**) heart waveforms, the frequency in (**d**) to (**h**) is 60 Hz. Source data are provided as a Source Data file.

Based on the excellent electrochemical performance, the ACPEC unit was integrated into a model AC line filtering circuit to verify its filtering capability (Fig. 3a). Generally, the high-voltage AC signal from the power plant is firstly converted into the rated low-voltage AC signal required by the electronic devices through the transformer, which is rectified into a pulsating DC signal through the rectifier. And then a stable DC signal is obtained through filtering capacitors. During the filtering process, the capacitor is just like a reservoir, in which no matter how the magnitude of the input signal changes, the output signal can still be steady (Supplementary Fig. 23a). If the input voltage is higher than that of the capacitor, the capacitor is charged. On the contrary, the capacitor will release charges to maintain the voltage stability of the circuit. (Supplementary Fig. 23b, c). First of all, the filtering performance of ACPEC is evaluated with the load resistance of 1 kΩ under 25 °C. Benefiting from the large capacitance and excellent frequency response performance, the ACPEC (287 μF at 120 Hz) exhibits a ripple voltage of 23 mV, smaller than that of AEC (50 mV, 220 μF/16 V, CHONGX, China, Supplementary Fig. 24). At the same time, the ripple voltage of ACPEC reaches 75 mV under the maximum allowable operating temperature of 105 °C (Supplementary Figs. 25, 26). And the corresponding maximum ripple current of ACPEC is calculated as 75 μA, which is higher than that of AEC (67 μA, 220 μF/16 V, CHONGX, China). In addition, to optimize the output voltage of ACPEC unit, the load of 10 MΩ is chosen to connect into circuit (Supplementary Fig. 27). Interestingly, one ACPEC unit could convert a sinusoidal AC waveform signal (3.2 V_peak-peak_, 60 Hz) into smooth DC signal with only 10 mV fluctuations, which is 13 times smaller than that of the AEC (130 mV, 220 μF/16 V, CHONGX, China) with a similar capacitance (Fig. 3b). This is further verified by 180 parallel tests with oscilloscope (RTB2002, Rohde & Schwarz, Germany, Supplementary Fig. 28a, b). Comparing with that of AEC (220 μF/16 V, CHONGX, China), the smoother DC output signal of ACPEC unit may be benefited from its smaller ESR and relaxation time constant (*τ*_0_) (Supplementary Fig. 29a, b). Besides, the frequency compatibility is also critical for filtering capacitors in practical application. As shown in Fig. 3c and Supplementary Fig. 30, even if the input AC waveform signals are at the frequency range of 1~10,000 Hz, the output signals still present smooth line shape with negligible variances below 0.01, smaller than those of AEC (almost 0.08, 220 μF/16 V, CHONGX, China). The result can be explained with the lower total impedance of the ACPEC unit over the frequency range of 1~10^5^ Hz (Supplementary Fig. 31), thus reducing energy loss, improving efficiency, and decreasing ripples below self-resonant frequency of ACPEC (~10 kHz). Furthermore, except for sinusoidal waveform, ACPEC can also filter other AC waveforms at 60 Hz, such as rounds PM, electrocardiogram, diamond, Lorentz and heart waveforms (Fig. 3d–h and Supplementary Fig. 32). These superb filtering properties of the ACPEC unit are mainly attributed to its large areal specific capacitance and ideal capacitive behavior and small dissipation factor, which are also the fundamental basis for obtaining excellent filtering performance of integrated devices.

### Flexibility of integrated filtering device with 7 ACPEC units

**Fig. 4:**
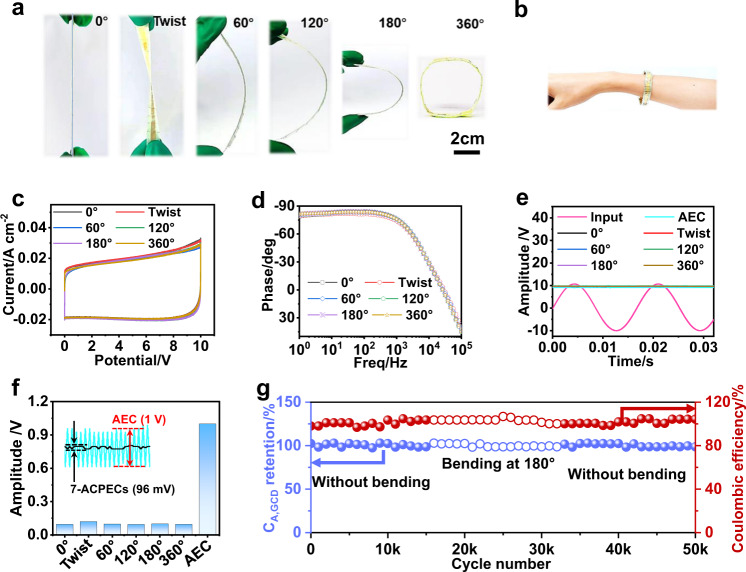
The flexibility and filtering performance of 7-ACPECs. **a** Photos of 7-ACPECs twisted and bent at different angles, such as, 0°, 60°, 120°, 180°and 360°, the scale bar is 2 cm. **b** Photo of 7-ACPECs being bent and worn on the wrist like a bracelet. **c** CV curves of 7-ACPECs under different deformation states (scan rate: 10 V s^−1^). **d** Plots of phase angle versus frequency of 7-ACPECs under different deformation states. **e** The filtering performance of 7-ACPECs under different deformation states. **f** The comparison of fluctuation amplitude of the output signals for 7-ACPECs under different deformation states with the commercial AEC. Inset shows the fluctuation signals of 7-ACPECs (0°) and AEC (47 μF/ 16 V, CHONGX, China). **g** The stability of the 7-ACPECs at the initial state (0°) for 15,000 cycles, and under bending at the angle of 180° for 20,000 cycles, then restoring to the initial state (0°) for 15,000 cycles. Source data are provided as a Source Data file.

Flexibility of devices contributes to the volume reduction and facilitates their integration in circuits at some extent. The 7-ACPECs is assembled with OAS strategy exhibits excellent flexibility (Fig. 4a). At the initial state, 7-ACPECs presents a strip-shape with the length of 7 cm, which can be easily twisted and bent without sacrificing its integrity, and even curled just like a portable bracelet (Fig. 4b). The flexibility of 7-ACPECs is further clearly observed from the electrochemical characterizations (Fig. 4c–e). As displayed in Fig. 4c, the 7-ACPECs exhibits highly overlapped CV curves and negligible changes of capacitance no matter at the initial state of 0° or the bending state of 360°. This is also verified by EIS test. All the Bode plots of 7-ACPECs under different deformations are similar (Fig. 4d). There is a slight reduction of phase angle (−81.4°) under twisting state at 120 Hz compared with the initial stable (−83°), probably due to the larger strain generated by the twisting. And all of the ESR, *τ*_0_, and *C*_120_ of the 7-ACPECs are almost constant under various deformations, Besides, the dissipation factor at 120 Hz (*DF*_120_) of 7-ACPECs (0°) is 10.6% (Supplementary Table S2), which is steady with single ACPEC unit (10.7%). These demonstrate the feasibility of OAS strategy and the flexibility of 7-ACPECs (Supplementary Fig. 33).

In addition, the AC line filtering performance of the 7-ACPECs under different deformation states was investigated, which could filter the sinusoidal AC signal (21 V_peak-peak_, 60 Hz) into smooth DC signal of ~9.5 V (Fig. 4e). The average amplitude of output signals from 7-ACPECs (~10 V, 40 μF) under different deformation states is about 96 mV, which is approximately 10 times less than that of AEC (47 μF/16 V, CHONGX, China) of 1 V (Fig. 4f). This is also verified by 180 parallel tests with oscilloscope (RTB2002, Rohde & Schwarz, Germany, Supplementary Fig. 34). It can be ascribed to the highly conductive and porous interconnected structure of electrodes, and the flexible characteristics of seamless connection between the adjacent ACPEC units, which endow 7-ACPECs with superior electrochemical performance to AEC (47 μF/16 V, CHONGX, China) (Supplementary Fig. 35), thus leading to a smoother output signal. Similar with the single ACPEC unit, 7-ACPECs can also smooth arbitrary AC waveforms into DC signals, indicating excellent waveforms adaptability (Supplementary Fig. 36). Moreover, the 7-ACPECs shows similar capacitance retention and coulombic efficiency under initial (0°) and bending state (180°) at the current density of 5 mA cm^−2^ over 50,000 cycles (Fig. 4g), indicating the excellent mechanical flexibility and durability of the integrated device.

### Scalable integration and application of ACPECs

**Fig. 5:**
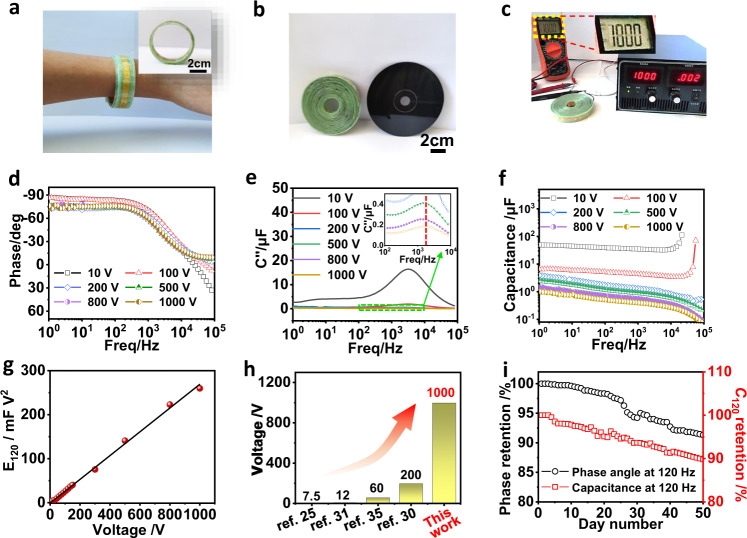
Filtering capacitors integrated with ACPECs in large-scale. The photographs of (**a**) 67-ACPECs, and (**b**) comparing the size of scrolled 670-ACPECs with a disk. **c** Photograph of 670-ACPECs charged to 1000 V at the current density of 8 mA cm^−2^. **d**−**f** The electrochemical performance of filtering capacitors integrated with various number of ACPECs. **d** Plots of phase angle versus frequency. **e** Plots of the imaginary part of capacitance (*C*”) versus frequency, inset: the frequency corresponding to the maximum *C*” of 340-ACPECs (green dotted line), 540-ACPECs (purple dotted line) and 670-ACPECs (yellow dotted line). **f** Plots of capacitance versus frequency of filtering capacitors integrated with different numbers of ACPECs in-series. **g**) Plot of *E*_120_ versus working voltage of ACPECs devices. **h** Comparing the maximum working voltage of the 670-ACPECs with the reported integrated ones^25,30,31,35^. **i** The retention of phase angle and capacitance (*C*_120_) of 670-ACPECs for 50 days. Source data are provided as a Source Data file.

With superior structural stability and high consistency, the filtering capacitors consisting of various number of ACPEC units can be simply integrated by using OAS strategy to meet practical AC line filtering with wide operating voltage ranges. The 67-ACPECs can be scrolled into a portable wristband (Fig. 5a), which can also exhibit waveform adaptability, and smooth the arbitrary AC waveforms to DC signals (such as sine, square, triangle and electrocardiogram waveforms at 60 Hz in Supplementary Fig. 37). After integrating with the rotating disk triboelectric nanogenerator (RD-TENG, Supplementary Figs. 38–40), a steady DC signal of 100 V can be output (Supplementary Fig. 40b). Particularly, the scrolling process can continue with the increment of units connected in series. Even with 670 ACPEC units, a scroller-like AC line filtering device (670-ACPECs) can be fabricated with the radius of only 4 cm, which is smaller than that of a portable disk (radius is 6 cm, Fig. 5b). An ultrahigh voltage of 1,000 V can be achieved at a constant current density of 8 mA cm^−2^ (Fig. 5c), higher than those integrated filtering capacitors reported previously^25,30,31,35^.

It is worth noting that the frequency responsiveness of the 670-ACPECs exhibit excellent stability even with amounts of units connected in series, as evident from EIS test. The results show that the phase angle and *τ*_0_ are almost similar with increasing the number of ACPEC units connected in series (Fig. 5d, e). Compared with the single ACPEC unit (–83.3°), the phase angle of 670-ACPECs at 120 Hz shows a slight decrease of 8.8 % (–76°, Fig. 5d), which is still comparable to that of many reported filtering capacitors^11,14,15,17,34,50–52^, indicating that the integrated high-voltage ECs have excellent high-frequency performance. In addition, the n-ACPECs, such as 340-ACPECs, 540-ACPECs, and 670-ACPECs display similar relaxation time *τ*_0_ (~0.48 ms) with that of the single ACPEC unit (~0.46 ms, Fig. 5e), mainly attributed to the flexibility and stability of film electrodes, the close adhesion between the electrodes and the current collectors, and the seamless connection between the individual units. The Nyquist diagram of integrated ACPECs reveals almost vertical line at low frequency, indicating a pure capacitive behavior (Supplementary Fig. 41)^4^. In addition, even if 670 units are connected in series, the *C*_120_ and *E*_120_ of the integrated 670-ACPECs can reach about ~0.5 μF (Fig. 5f) and 250 mF V^2^ (69.5 μW h), respectively. For the thousand-volts of 670-ACPECs, it demonstrates greater advantageous in a wide voltage range, comparing to some commercial AECs with similar capacitance (e.g., 2.2 μF/450 V, Tinkersphere, USA; 2.2 μF/450 V, Samwa, Korea; 2.2 μF/450 V, Jamicon, China (Taiwan); 2.2 μF/450 V, Rubycon, Japan; 1 μF/400 V, GANGHUI, China; 1 μF/450 V, Jamicon, Europe (Prague); 1μF/450 V, KELTRON, India), which can be expected to be applied in AC line filtering that requires wider operating voltage ranges, such as complementary component in the power converter^53,54^. All of the above parameters demonstrate the high-rate performance of integrated ACPECs device. Besides, *E*_120_ of integrated devices increases with the increase of working voltage (*E*_120_∝*V*). From Fig. 5g, *E*_120_ of integrated devices exhibit the linear relationship with the working voltage, implying the high compatibility and structural stability of the units within 670-ACPECs. The comparison of working voltage for 670-ACPECs in this work is also illustrated in Fig. 5h and Supplementary Table S3. At the same time, the integrated n-ACPECs devices show higher volumetric advantages at 120 Hz than AECs below ~100 V, and comparable volumetric specific capacitance at working voltage ~500 V, which are higher than the filtering capacitors reported previously (Supplementary Fig. 42)^5,7,17,25,30,52^.

Moreover, the 670-ACPECs exhibits excellent stability, which can be seen from the almost coincident impedance spectra before and after charge/discharge process (Supplementary Fig. 43). Meanwhile, the stability can also be observed from the approximately unchanged phase angle and capacitance retention rate of 670-ACPECs for 50 days. Both the phase angle and capacitance retention rate stabilize at 100% for the first three days. Even after 50 days, the phase angle retention rate is at 91.3%, and the capacitance retention rate is 91% (Fig. 5i). The excellent stability is attributable to the seamless integration process that makes each unit tightly connected and the double sealing process that alleviates the volatilization of the electrolyte (Supplementary Figs. 44, 45).

Furthermore, for the integrated 670-ACPECs, the ripple current is 67 mA under 25.8 ± 1 °C (Supplementary Table S2), which is comparable with some commercial products (e.g., Nichicon of 86 mA, and BERYL of 70 mA), implying the potential of high-voltage integrated devices for practical filtering. Owing to the rationally designed structure and the flexibility of electrodes, as well as the scalable assembly strategy, the ultrahigh working voltage of 1,000 V for AC line filtering is achieved for the first time, which presents a prospect to attenuate the leftover AC ripples in line-powered system with higher working voltage requirements.

## Discussion

This work reported an ACPEC with a large phase angle of –83.3°, a short *τ*_RC_ of 0.15 ms and an ultra-high *E*_A, 120_ of 1.29 mF V^2^ cm^−2^ (0.36 μW h cm^−2^), greater than that of the reported common aqueous filtering capacitors. Moreover, the ACPEC could smooth the ripples in wide frequency range from 1 to 10,000 Hz. The integrated device 7-ACPECs (~10 V, 40 μF) exhibited excellent flexibility and filtering performance with an ultralow fluctuation of 96 mV under 10 V, which is 10 times less than that of commercial AEC (47 μF/16 V, CHONGX, China) of 1 V. After integrating 67-ACPECs with the rotating disk triboelectric nanogenerator, a stable DC voltage signal of 100 V was output, endowing TENG with more potential to be applied as a power source in real life. Furthermore, the record-high working voltage of integrated filtering capacitors could reach up to 1,000 V. Nevertheless, the integration of high-voltage ACPECs in this work mainly relies on manual operation, automated operations including laser technology, 3D printing, etc. should be considered to achieve the fabrication of such devices in one step in the future. Above all, this work promotes the operating voltage of integrated filtering capacitors, which provides more possibilities for filtering capacitors to be applied in a variety of practical scenarios.

## Methods

### Preparation of positive CPN film electrode

A cellulose membrane (0.6 × 0.6 cm^2^) was used as template. Next, the mixed solution of PEDOT:PSS (Clevios PH1000, Heraeus) and 13.vol% DMSO was dropped on the cellulose membrane, followed by a spin-coating (500 rpm for 15 s and 2000 rpm for 10 s) treatment to ensure uniform and sufficient penetration of the mixed solution into the cellulose membrane. After drying in an oven at 65 °C for 20 min, the dried membrane was immersed in 18 M concentrated sulfuric acid for overnight at temperature of 25 °C, followed by rinsing in deionized water for multiple times. Then, the as-prepared wet continuous PEDOT nanomesh hydrogel film (CPN) was placed and attached on an Au foil (thickness: 50 μm). With the evaporation of water molecules in the film, the CPN film gradually adhered closely to the Au substrate through intermolecular forces. Finally, the positive CPN film electrode on Au foil can be obtained after drying in an oven (60 °C, 1~2 min) and trimming the CPN film into the shape of 0.5 × 0.5 cm^2^ (Supplementary Fig. 7a). The thickness of the electrodes could be modulated by changing the dropping volume of the PEDOT:PSS/DMSO mixing solution. And the different CPN films were named as CPN-n (n = 90, 200, and 500 nm, the thickness of CPN film).

### Preparation of negative p-CNT film electrode

The p-CNT film was prepared by H_2_O_2_ etching and mechanically peeling process. The CNT paper was purchased from Nanjing Xianfeng Co., LTD and used directly without further treatment. In a simple procedure, the CNT paper was placed in H_2_O_2_ (30%), and refluxed at 70 °C more than 15 h to obtain CNT hydrogel with porous structure. Then, after soaking it in deionized water for several times to remove the residual H_2_O_2_, the p-CNT film was obtained by mechanically peeling. Specifically, a desired thickness of p-CNT film can be obtained by mechanically peeling with pointed tweezers from the CNT paper after H_2_O_2_ etching and deionized water washing. The thickness of film was verified by cross-sectional characterization of SEM (Supplementary Fig. 2d–f). The exfoliated wet thin p-CNT film was directly placed and attached on Au foil without any adhesive. With the evaporation of water molecules in the film, the electrode films gradually and closely adhered to the Au substrate through intermolecular forces. After fully dried in an oven at 60 °C for 1~2 min and trimmed into a square shape with a size of 0.5 × 0.5 cm^2^, the negative p-CNT film electrode can be acquired (Supplementary Fig. 7b). The thickness of the electrodes could be modulated during mechanically peeling. The p-CNT films with different thickness were named as p-CNT-n (*n* = 90, 200, and 500 nm, the thickness of CNT films).

### Electrochemical characterizations of single electrodes

The CPN and p-CNT were characterized by the three-electrodes system, respectively. The Pt wire, Ag/AgCl (saturated KCl) electrode, and 3 M H_2_SO_4_ were served as the counter electrode, reference electrode, and electrolyte, respectively.

### Fabrication of an ACPEC unit

An ACPEC unit was fabricated by assembling CPN positive electrode and p-CNT negative electrode in a sandwiched manner, and sealed with parafilm. Specifically, CPN and p-CNT film electrodes (each with an area of 0.5 cm × 0.5 cm) were assembled in a two-electrode configuration using cellulose diaphragm (NKK, Japan, TF4050, thickness of 40 μm) as a separator, 3 M H_2_SO_4_ as the electrolyte, and Au foils (thickness of 50 μm) as current collectors. The electrochemical performance of ACPEC could be controlled with adjusting the materials loading of electrodes. Different ACPEC units were named as ACPEC-n (*n* = 90, 200, and 500 nm, the thickness of electrodes materials).

### Fabrication of integrated filtering capacitors with various numbers of ACPEC units

The integrated filtering capacitors were assembled with the OAS strategy. Specifically, CPN film and p-CNT film were attached onto the same side of the two ends of each Au foil current collector as positive and negative electrodes, respectively (Supplementary Fig. 8), which was attached on the thermal release tape with high viscosity for a tight fit. Then, the CPN positive electrode of Au foil was assembled with p-CNT negative electrode of another Au foil to form a single ACPEC unit. In a similar strategy, a strip-shaped integrated device was easily obtained by sequentially assembling ACPEC units on one piece of high-viscosity thermal release tape, pressing another piece of high-viscosity thermal tape against the other side of the integrated device for bonding, and wrapping with BOPP tape again. Various numbers of the ACPEC units were connected in series as required. Finally, the flexible integrated filtering capacitors can be easily obtained by scrolling the integrated ACPEC units like a scroller. The capacitors can be bent and twisted without sacrificing their integrity.

### Electrochemical characterizations of two-terminal devices

The EC was tested by using a CHI 660E Potentiostat (CH Instruments Inc., China). For Cyclic voltammetry (CV) and galvanostatic charge-discharge (GCD) measurements, the electrochemical windows were controlled to be 0 − 1.5 V. EIS tests were performed in the frequency range of 10^5^ to 1 Hz at an amplitude of 5 mV and bias voltage of 0 V. CV tests of connected ECs in series or in parallel were carried out by using a Keithley 2450. For the AC-line filtering test, all the input AC signals were supplied by an arbitrary function generator (33511B, Agilent Technologies Inc., Tektronix, USA). For the AC line filtering test of connected ECs, the input signals were enlarged by an ATA-2041 high-voltage amplifier (Agitek, China). All the outputs were recorded by a RTB2002 mixed domain oscilloscope (Rohde & Schwarz, Germany). The above tests were performed in ambient temperature of 25 °C.

### Parameter calculations

In order to avoid overcharging of any electrode in the device, the load of the active materials of the positive electrode and the negative electrode were adjusted according to the charge balance Eqs. (1, 2)^30^:

1 $${q}_{+}={q}_{-}$$

2 $${C}_{A+}\times \varDelta {V}_{+}={C}_{A-}\times \varDelta {V}_{-}$$

where *q* is the charge stored in the electrode, *C*_A+_ is the capacitance per unit area of the positive electrode, *C*_A-_ is the capacitance per unit area of the negative electrode, Δ*V* is the voltage amplitude.

The capacitance (*C*), areal specific capacitance (*C*_A_), resistance-capacitance time constant (*τ*_RC_), the volumetric specific capacitance of the device at 120 Hz (*C*_V, device, 120_), the dissipation factor at 120 Hz (*DF*_120_), CV/volume of the single electrode at 120 Hz (CV/volume_120_), the real and imaginary areal specific capacitances (*Cʹ* and *C″*, respectively), relaxation time constant (*τ*_0_), the areal specific energy density (*E*_A_) and energy (*E*) of the ACPEC were calculated using Eqs. (3–12), respectively^12,22,30^:

3 $$C=-\frac{1}{2\pi fZ^{\prime\prime} }$$

4 $${C}_{A}=-\frac{1}{2\pi fZ^{\prime\prime} S}$$

5 $${\tau }_{RC}=-\frac{Z^{\prime} }{2\pi fZ^{\prime\prime} }$$

6 $$D{F}_{120}=\,\tan \sigma \times 100\%=-\frac{Z^{\prime} }{Z^{\prime\prime} }\times 100\%$$

7 $${C}_{V,device,120}=\frac{{C}_{120}}{volum{e}_{device}}$$

8 $$CV/volum{e}_{120}=\frac{{C}_{120}\times U}{volum{e}_{device}}$$

9 $$C^{\prime}=-\frac{Z^{\prime\prime} }{2\pi f|Z{|}^{2}S}\,C^{\prime\prime}=-\frac{Z^{\prime} }{2\pi f|Z{|}^{2}S}$$

10 $${\tau }_{0}=\frac{1}{{f}_{0}}$$

11 $${E}_{A}=\frac{1}{2}\times {C}_{A}\times {U}^{2}$$

12 $$E=\frac{1}{2}\times C\times {U}^{2}$$

In the Eqs. (3–12), *f* is the frequency; *Z*′ and *Z*″ are the real and imaginary impedance, respectively. *S* is the area of electrode; tan*σ* is the loss tangent, *σ* is the angle in a complex plane of Nyquist plot. *C*_120_ is the total capacitance of n-ACPECs device at 120 Hz. The volumes of the n-ACPECs device are listed in Table S2, the thickness including the current collects, electrode materials, separator, and the uncompacted gaps. *f*_0_ is the frequency at maximum *C”*. *E* is the energy of n-ACPECs. *C* is the capacitance of n-ACPECs device. *U* is the voltage window of n-ACPECs. The n is the number of the ACPEC units.

The capacitance of ACPEC from the discharge curves of galvanostatic charge-discharge test (*C*_*A,GCD*_) was also calculated by using Eq. (13):

13 $${C}_{A,GCD}=\frac{I\varDelta t}{S\varDelta V}$$

where *I* is the constant discharge current, Δ*t* is the discharge time, and Δ*V* is the discharge voltage drop (excluding IR drop).

### Measurement of the ripple current at temperature of 25 °C

The circuit is as the same as that for the AC-line filtering test. To obtain the ripple current at 25 °C and load, the input AC voltage signals were supplied by a generator with a frequency of 60 Hz. The voltage outputs were recorded by a MSO5104 mixed domain oscilloscope (Tektronix, USA). The current outputs were recorded by a K2 current clamp (Chauvin Arnous, France). The temperatures were measured by a FOTC-MINA-001800-N fiber optic temperature probe (Indigo, China).

### Fabrication of the rotating disk triboelectric nanogenerator (RD-TENG)

According to the design sizes, the acrylic sheets (2 mm thick) are directly purchased from Shanghai Lujuan Trading Co., Ltd. as the supporting substrate. The customized copper foil and polytetrafluoroethylene (PTFE) are firmly and evenly attached to the acrylic sheets, so that the surfaces of the copper foil and the PTFE film are parallel to each other (Supplementary Figs. 34–36)^31^.

### Electrical output measurement of the RD-TENG

In the electrical output measurement, the PTFE part of the RD-TENG was bonded onto a powerful direct current motor (997 high speed), and the copper part was secured, with both centers of the disks in coincidence with the spinning axis. The PTFE part was driven to rotate around the axis of the motor. The open-circuit voltage and accumulated charge was measured by Keithley 6514 system.

### Characterization

Field-emission scanning electron microscopy (FE-SEM) was performed using a JSM-7001F SEM (Japan Electron Optics Laboratory Corporation, Japan). The morphology of samples was also characterized by field-emission transmission electron microscope (FE-TEM) with high-angle annular dark-field (HAADF) detector (Tecnai G2 F20 U-TWIN) operating at 200 kV. Electrical conductivity was measured by a four-point technique via a four-point probe KDY-1 sheet resistivity tester (Kunde Technology, Guangzhou, China). Raman spectra were collected using a LabRAM HR Evolution Raman spectrometer (HORIBA Jobin Yvon, France) with a 532 nm laser. X-ray photoelectron spectroscopy (XPS) data were collected using an Escalab 250 photoelectron spectrometer (ThermoFisher Scientific, USA) using 300 W Al Kα irradiation. The thickness of the conductive film was measured by SEM images. Nitrogen adsorption-desorption experiments were conducted at 77 K using a TriStarII3020 apparatus (Micromeritics Instrument Corporation, USA). Before the adsorption measurements, the negative electrode p-CNT film and its contrast samples were degassed in vacuum from 300 K to 403 K at heating rate of 2 °C min^−1^, and held at 403 K for 5 h. The specific surface areas were calculated by Brunauer-Emmett-Teller (BET) analyses of their adsorption isotherms. The high working voltage across the devices connected in-series was measured by HKY-1000E and Keithley 2450 system.

## Supplementary information

Supplementary Information

Description of additional Supplementary File

Supplementary Movie 1

Supplementary Movie 2

## Data Availability

The relevant datasets generated and analyzed in this study are provided with this paper. Source data are provided with this paper.

## Source data

Source Data
