# Supplementary material for: Aqueous hybrid electrochemical capacitors with ultra-high energy density approaching for thousand-volts alternating current line filtering
Source: Nat Commun. 2022 Oct 26;13:6359. doi: 10.1038/s41467-022-34082-2 (PMC9606111; doi:10.1038/s41467-022-34082-2)
Supplement: Supplementary file 1 — Supplementary Information [file 41467_2022_34082_MOESM1_ESM.docx]

**Supplementary Information**

**Aqueous hybrid electrochemical capacitors with ultra-high energy density approaching for thousand-volts alternating current line filtering**

**Li et al.**

**Supplementary Results and Discussion**


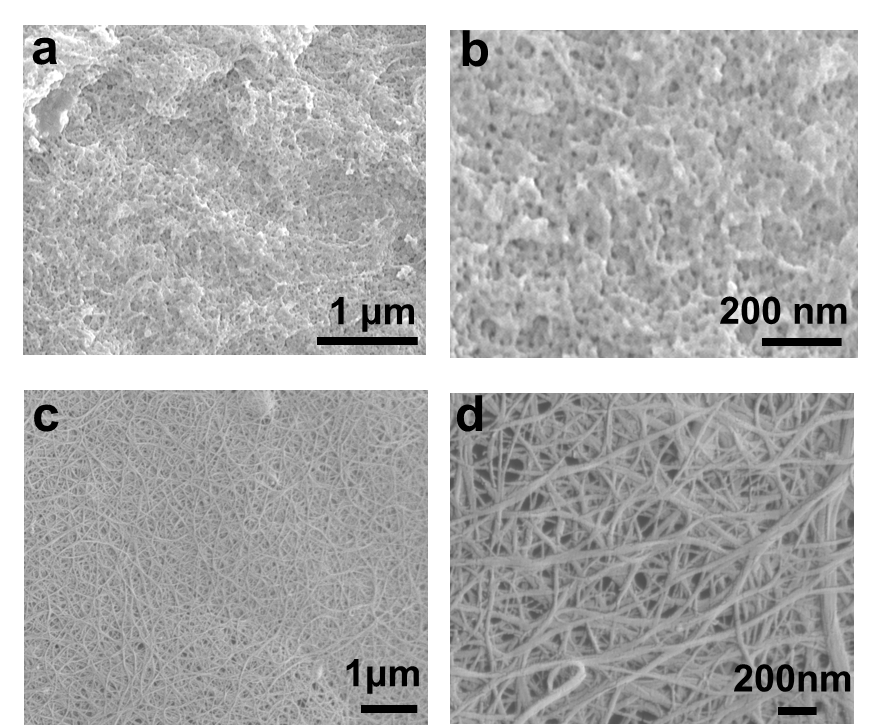


**Figure S1.** (a, b) The SEM images of the continuous PEDOT:PSS nanomesh (CPN) film under different magnifications. (c, d) The SEM images of the porous carbon nanotube (p-CNT) film under different magnifications.


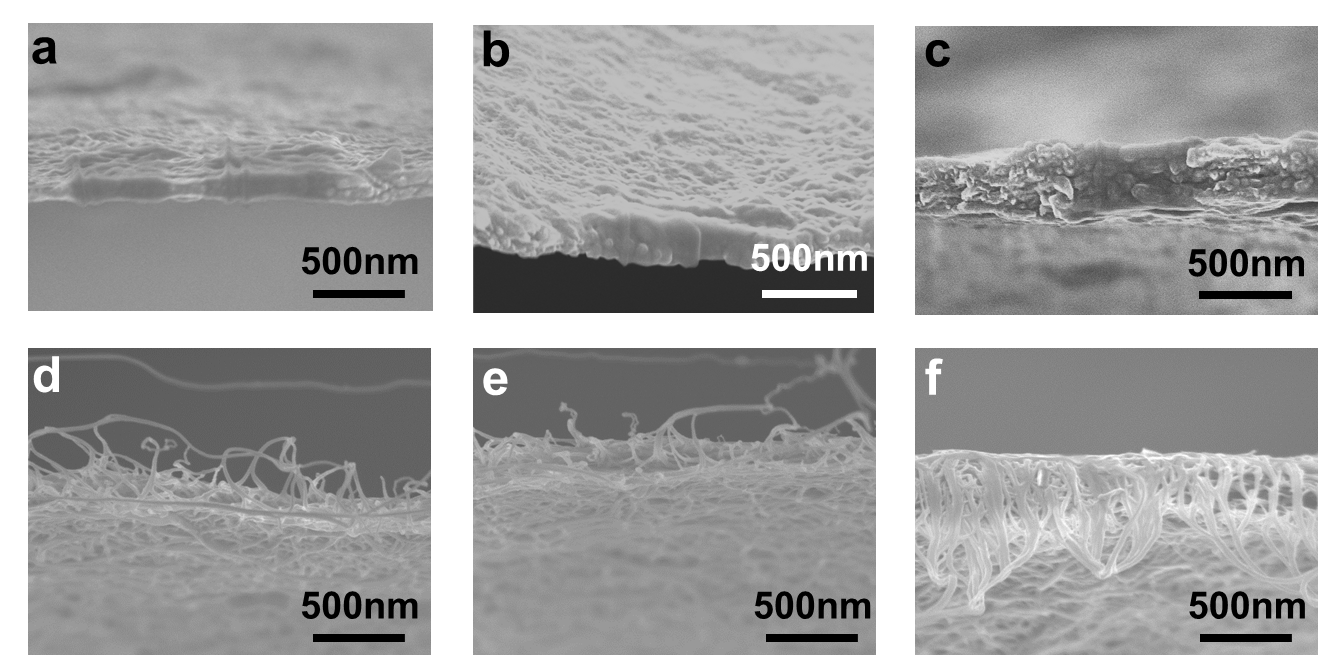


**Figure S2.** The SEM images of the cross section of (a) CPN-90, (b) CPN-200, (c) CPN-500, (d) p-CNT-90, (e) p-CNT-200, (f) p-CNT-500 film electrodes.

As shown in Figure S2 (a-c), the thickness of CPN film can be modulated by changing the dropping volume of the PEDOT:PSS/DMSO mixing solution. The CPN films with different thicknesses of approximately 90 nm (Figure S2a), 200 nm (Figure S2b) and 500 nm (Figure S2c) are confirmed by the SEM characterizations, which are called as CPN-n (n = 90, 200, and 500 nm). Besides, the thickness of p-CNT film can be controlled by mechanically peeling with pointed tweezers in deionized water. The p-CNT films with different thicknesses of approximately 90 nm (Figure S2d), 200 nm (Figure S2e) and 500 nm (Figure S2f) can be observed in SEM, which are named as p-CNT-n (n = 90, 200, and 500 nm).


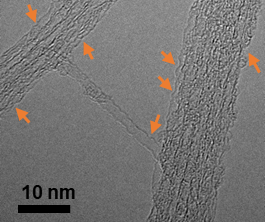


**Figure S3.** The TEM image of p-CNT film.

As shown in **Figure S3**, p-CNT films exhibit rough and curved tube walls with decoration of pore defects after H_2_O_2_ etching treatment and mechanically peeling.


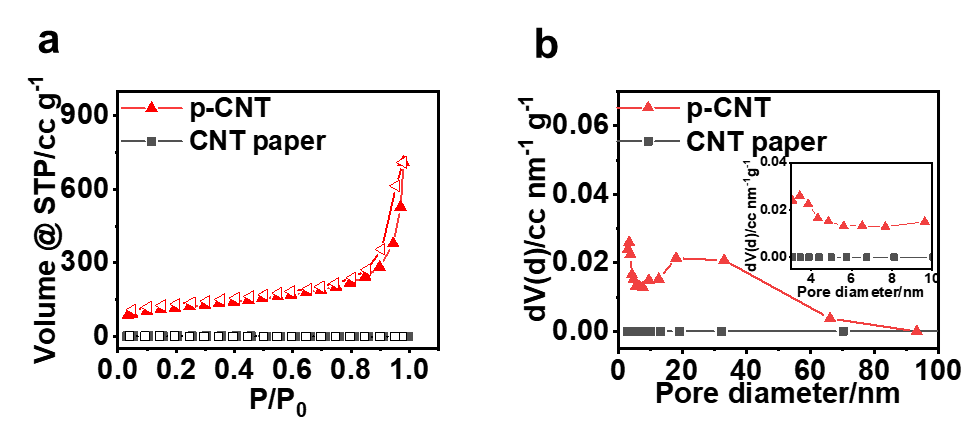


**Figure S4.** N_2_ absorption/desorption test results of pristine CNT paper and p-CNT film. (a) The specific surface area and (b) the pore diameter distribution range. Source data are provided as a Source Data file.

The results of N_2_ absorption/desorption test in Figure S4 show that the specific surface area of the p-CNT film is remarkably increased to 207.1 m^2^/g in comparison with that of pristine CNT paper (0.1 m^2^/g), which is mainly due to the existence of pore defects and loosely interconnected network of p-CNT film.


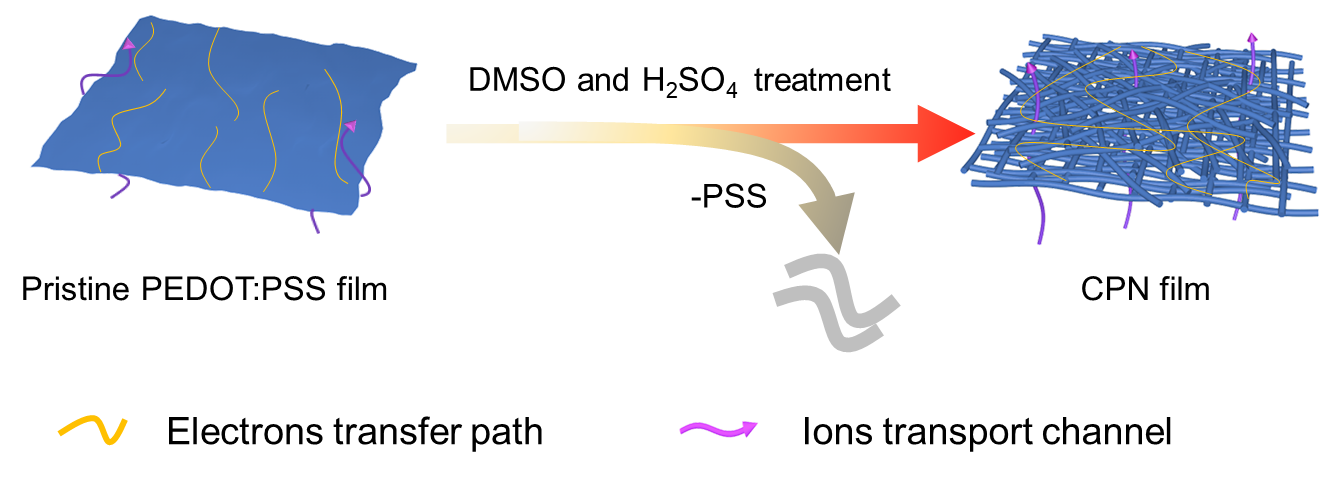


**Figure S5.** Schematic diagram of pristine PEDOT:PSS film after treatment.

As shown in Figure S5, the CPN film can be acquired after DMSO and concentrated H_2_SO_4_ treatment. The CPN film exhibits more continuous electrons transfer paths and more pores than the one before treatment, because the part of PSS can be removed and the PEDOT molecules rearrange, which are not only conducive to full contact between the active material and the electrolyte, but also promoting the charge transport, thereby the capacitance and rate performance of CPN electrode can be improved.


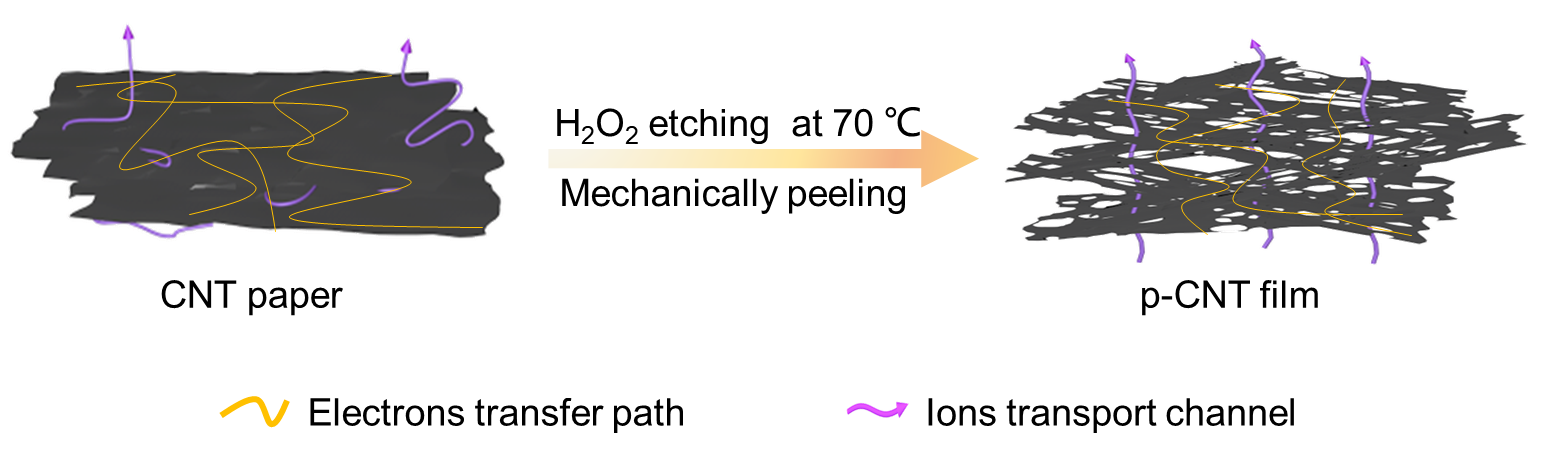


**Figure S6.** Schematic diagram shows CNT paper after H_2_O_2_ etching at 70℃ and mechanically peeling to obtain the p-CNT film.


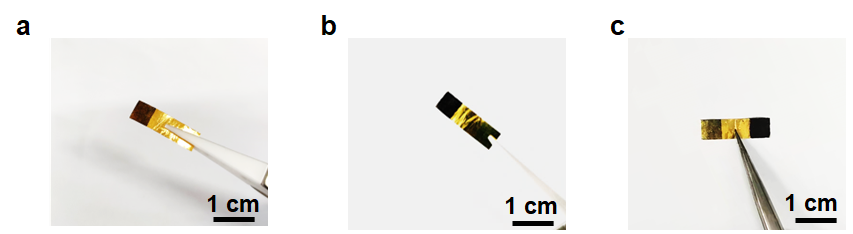


**Figure S7.** Optical images of CPN film positive electrode (a), the brown square is the CPN film on the Au foil. And the p-CNT film negative electrode (b), the black square is the p-CNT film on the Au foil.


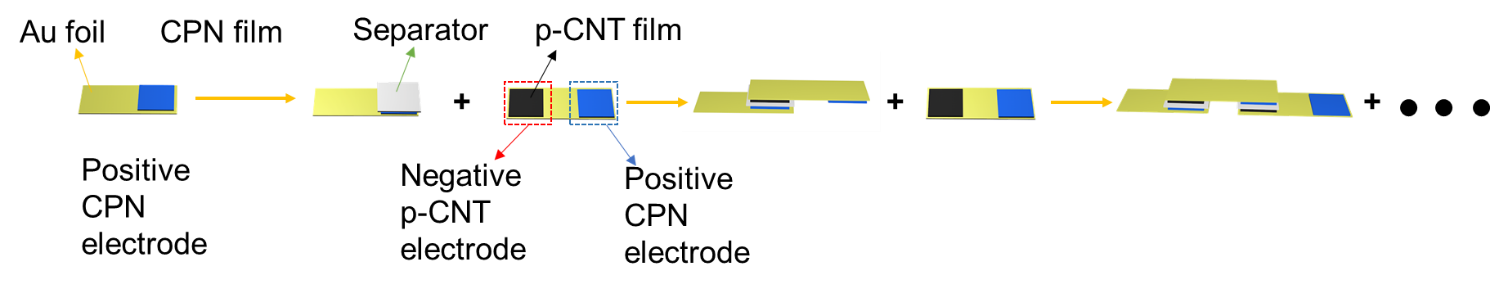


**Figure S8.** The detailed assembly process of integrated ACPECs.


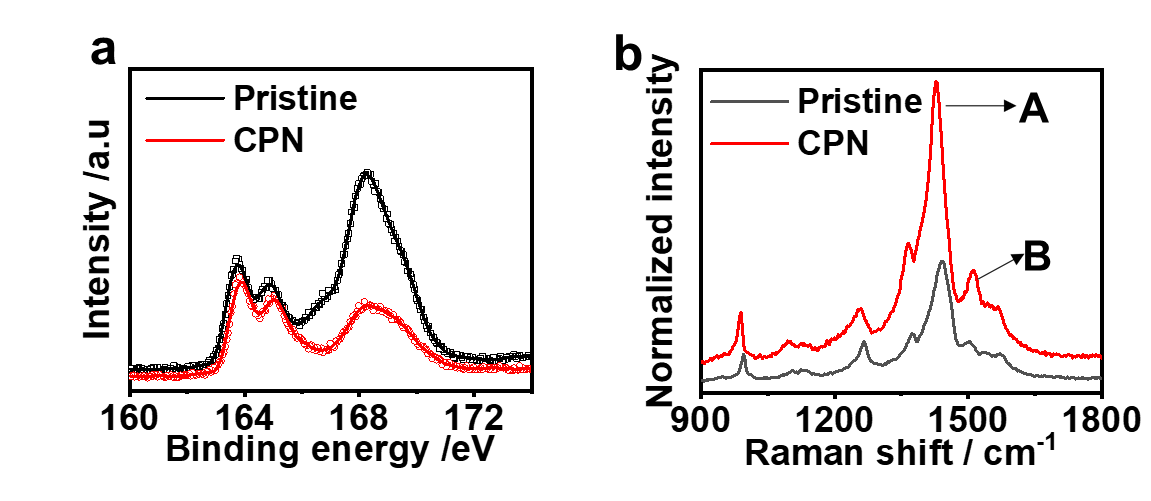


**Figure S9.** Chemical Structure of pristine PEDOT:PSS film and CPN film. (a) High resolution S2p XPS spectra and (b) Raman spectra of pristine PEDOT:PSS film and CPN film. Source data are provided as a Source Data file.

As shown in Figure S9a, for both pristine PEDOT:PSS film and CPN film, the XPS band between 166 and 172 eV is the S2p band of the sulfur atoms in PSS, whereas the two XPS bands between 162 and 166 eV are the S2p band of the sulfur atoms in PEDOT, respectively. As can be seen, the S2p intensity ratio of PEDOT to PSS significantly increases to 0.6 for CPN film after treatment, which is ~1.5 times higher than that of pristine PEDOT:PSS film (the ratio of PEDOT to PSS is 0.4). This indicates the successful removal of some PSS chains from the CPN film, which is important for enhancing the conductivity of CPN film, as suggested in the literature^1^. Besides, in the Raman spectra (Figure S9b), the bands at1427 cm^-l^ and 1511 cm^-l^ are assigned to the symmetric and anti-symmetric *C_α_*–*C_β_* stretching vibrations of thiophene rings, respectively. The intensity ratio of *I*_B(1511)_/*I*_A(1427)_ is further used to describe conjugation lengths of PEDOT chains. The larger the ratio, the longer the conjugation lengths^2,3^. The ratio of *I*_B(1511)_/*I*_A(1427)_ is 0.4 for CPN film, which is higher than that pristine PEDOT:PSS film (~0), indicating the increased conductivity of CPN film.


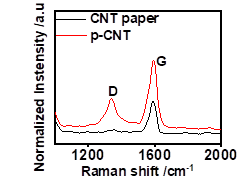


**Figure S10.** Raman spectra of CNT paper and p-CNT film. Source data are provided as a Source Data file.

As shown in Figure S10, there are two bands related to the C–C stretching mode of CNT, which are the G band at ~1590 cm^-1^ and D band near 1340 cm^-1^, respectively^4,5^. The *I*_D_/*I*_G_ ratio of CNT paper is ~0, while the *I*_D_/*I*_G_ value of p-CNT film increases to 0.54 after H_2_O_2_ etching and mechanically peeling, indicating that the increase of surface defects.


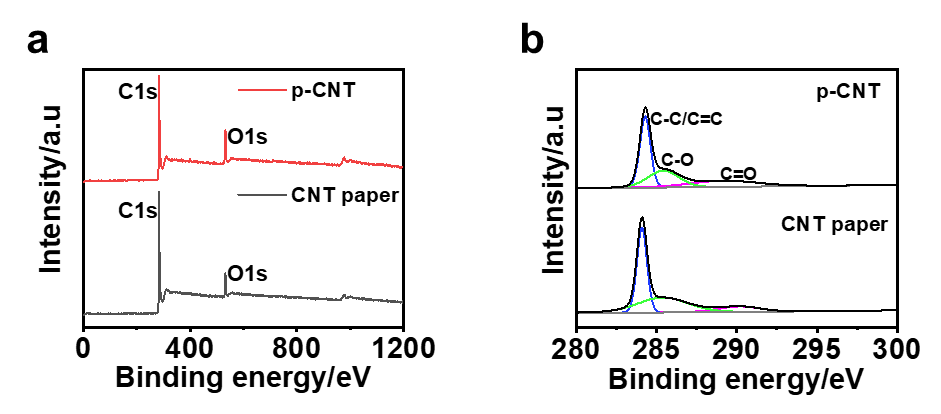


**Figure S11.** Chemical Structures of p-CNT film and pristine CNT paper. (a) XPS survey spectra of p-CNT film and pristine CNT paper. (b) High resolution C1s XPS spectra of p-CNT and pristine CNT paper. Source data are provided as a Source Data file.

The XPS examinations of oxygen and carbon atoms by the XPS surface chemical analysis show that the O/C ratio on the surface of pristine CNT paper and p-CNT film with H_2_O_2_ treatment are 0.07 and 0.11, respectively (Figure S11a). The result suggests that the oxygen content of the p-CNT film increases after H_2_O_2_ treatment, which is also conducive to the storage of electric charges. For XPS C1s spectra of p-CNT film (top) and CNT paper (bottom) (Figure S11b), the peaks at 284.3, 286.7 and 288.5V are corresponding to C–C/C=C, C–O (such as epoxy and alkoxy groups) and carboxyl functional groups, respectively. The C1s XPS spectrum clearly shows that the C–O functional group increases after treatment. This is also verified by the increased percentage of oxygen atoms, in which the oxygen content of CNT paper (atomic percentage) is 6.7% and increases to 9.7% after treatment.


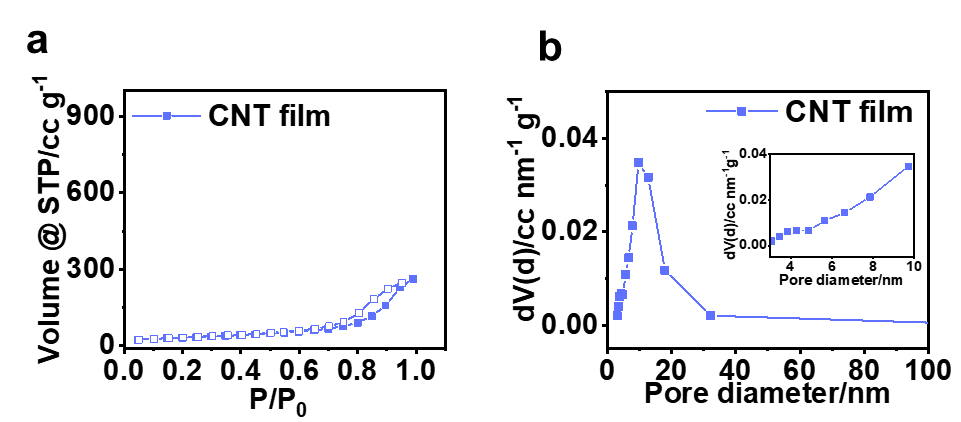


**Figure S12**. The N_2_ absorption/desorption test results of CNT film. (a) The specific surface area and (b) the pore diameter distribution range. Source data are provided as a Source Data file.

As shown in Figure S12a, the specific surface area of CNT film is 114.6 m^2^ g^-1^, which is about 1.8 times smaller than that of p-CNT film (207.1 m^2^ g^-1^, Figure S4), and the pore size distribution of CNT film is between 4.4~30 nm (Figure S12b).


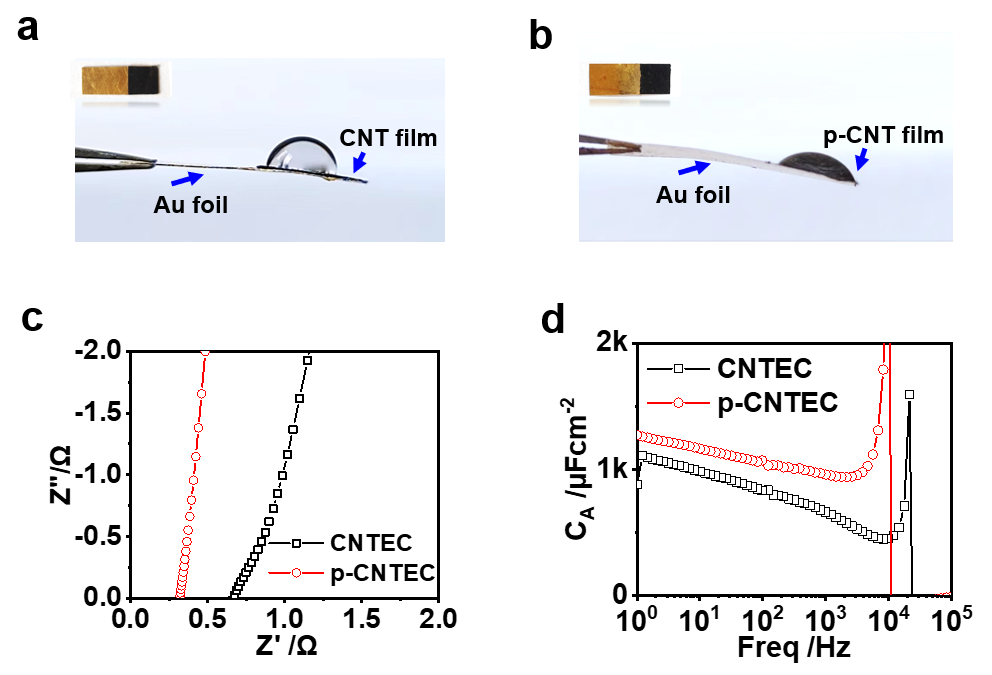


**Figure S13**. The comparison of hydrophilicity and electrochemical performance between CNT film-based symmetrical capacitor (CNTEC) and p-CNT film-based symmetrical capacitor (p-CNTEC). The water contact angles on the (a) CNT film electrode, and (b) p-CNT film electrode, which are attached on the Au foils. (c) Nyquist plots and (d) plots of *C*_A_ versus frequency of CNTEC and p-CNTEC, the sizes of CNT paper and p-CNT films are 0.5×0.5 cm^2^. Source data are provided as a Source Data file.


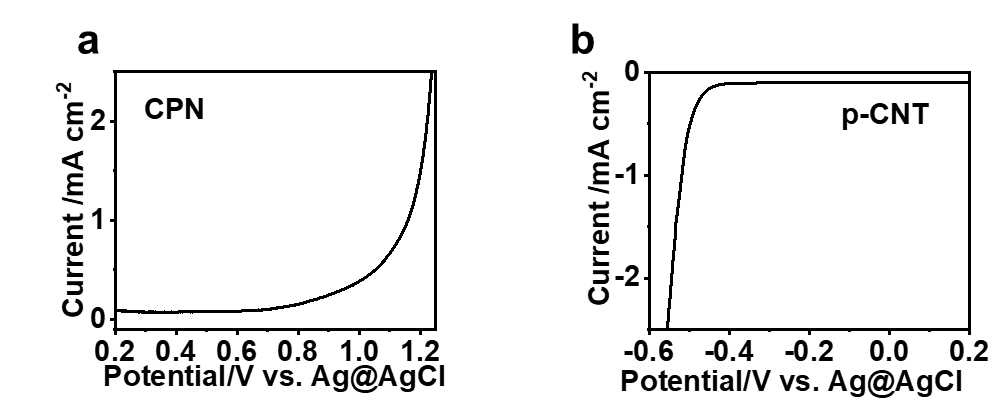


**Figure S14.** Linear sweep voltammetry (LSV) curves of the positive CPN film electrode (a) and negative p-CNT film electrode (b). Source data are provided as a Source Data file.

Positive CPN film electrode and negative p-CNT film electrode were tested in three-electrode system. 3 M H_2_SO_4_ was used as the electrolyte and the potential sweep rate was 1 mV s^-l^. The result demonstrates that the potential of oxygen evolution reaction (OER) is above 1.0 V *vs.* Ag@AgCl for the CPN electrode (a), and (b) potential of hydrogen evolution reaction (HER) is below –0.5 V *vs.* Ag@AgCl for p-CNT electrode.


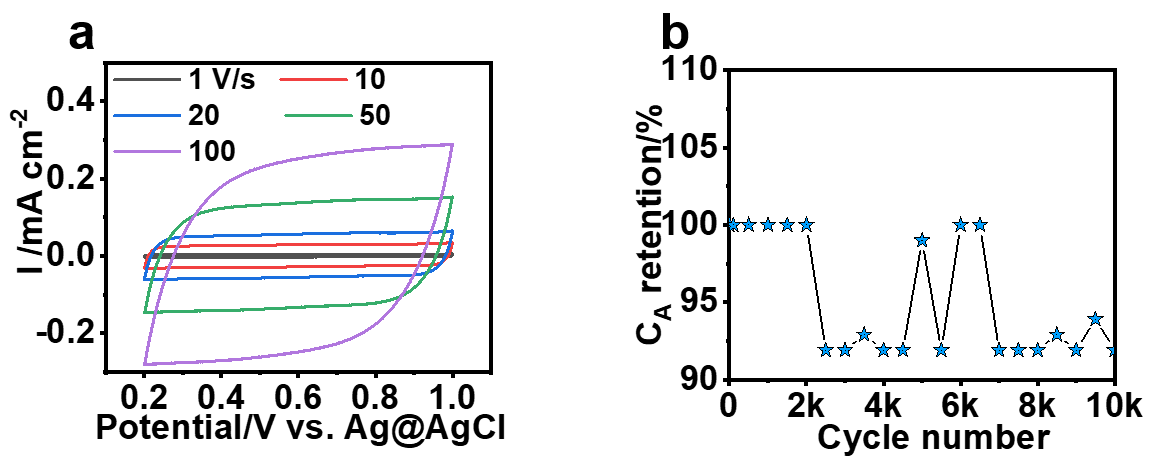


**Figure S15.** Electrochemical performance of CPN positive electrode in three-electrode system at 0.2~1.0 V *vs*. Ag@AgCl. (a) CV curves of CPN positive electrode within voltage window of 0.2~1.0 V *vs*. Ag@AgCl at different scan rates. (b) Cycling stability test of CPN positive electrode at 5 mA cm^-2^ in the voltage window of 0.2~1.0 V *vs.* Ag@AgCl for 10,000 cycles. Source data are provided as a Source Data file.


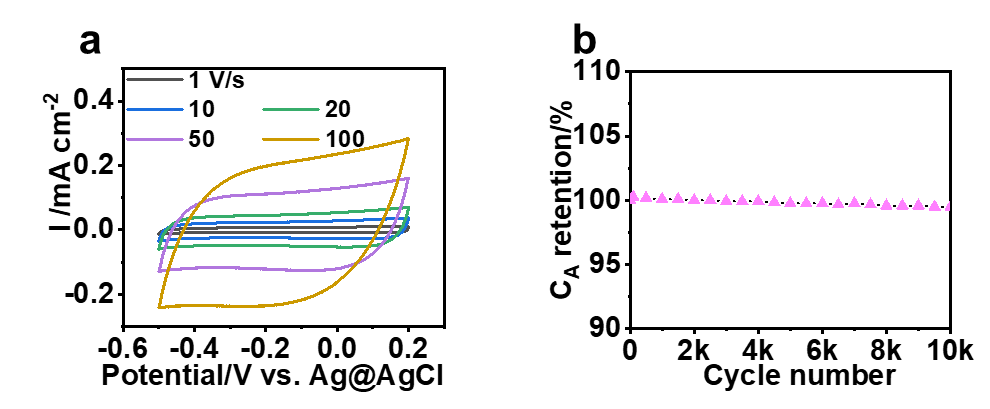


**Figure S16.** Electrochemical performance of p-CNT negative electrode in three-electrode system at –0.5~0.2 V *vs.* Ag*@*AgCl. (a) CV curves of p-CNT negative electrode within voltage window of –0.5~0.2 V *vs.* Ag*@*AgCl at different scan rates. (b) Cycling stability test of p-CNT negative electrode at the current density of 5 mA cm^-2^ in the voltage window of –0.5~0.2 V *vs*. Ag*@*AgCl for 10,000 cycles. Source data are provided as a Source Data file.


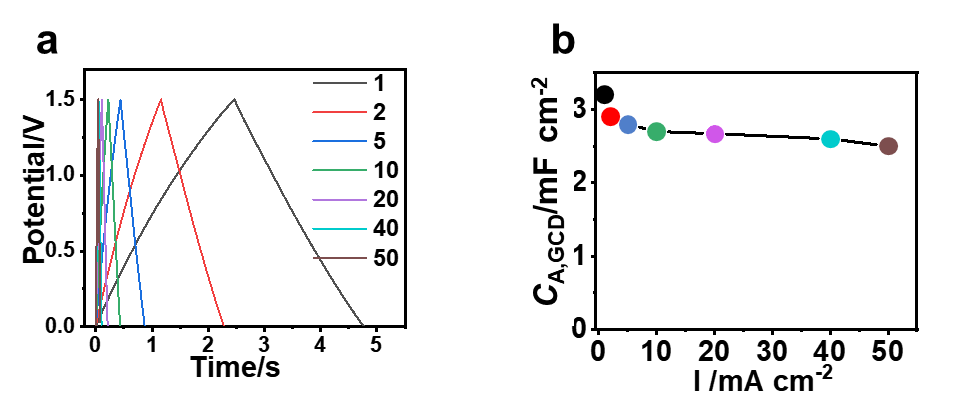


**Figure S17.** (a) The GCD test and (b) the areal capacitance of ACPEC at different discharge current densities from 1 to 50 mA cm^-2^. Source data are provided as a Source Data file.


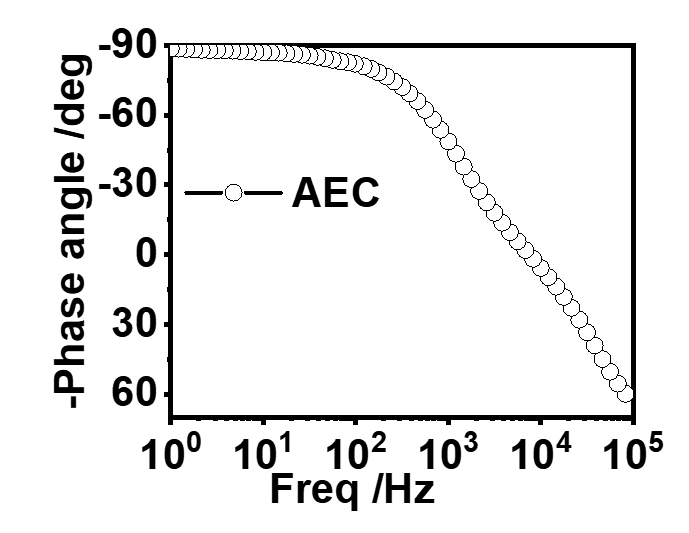


**Figure S18.** The phase angle of a commercial AEC (220 μF/16 V, CHONGX, China). Source data are provided as a Source Data file.


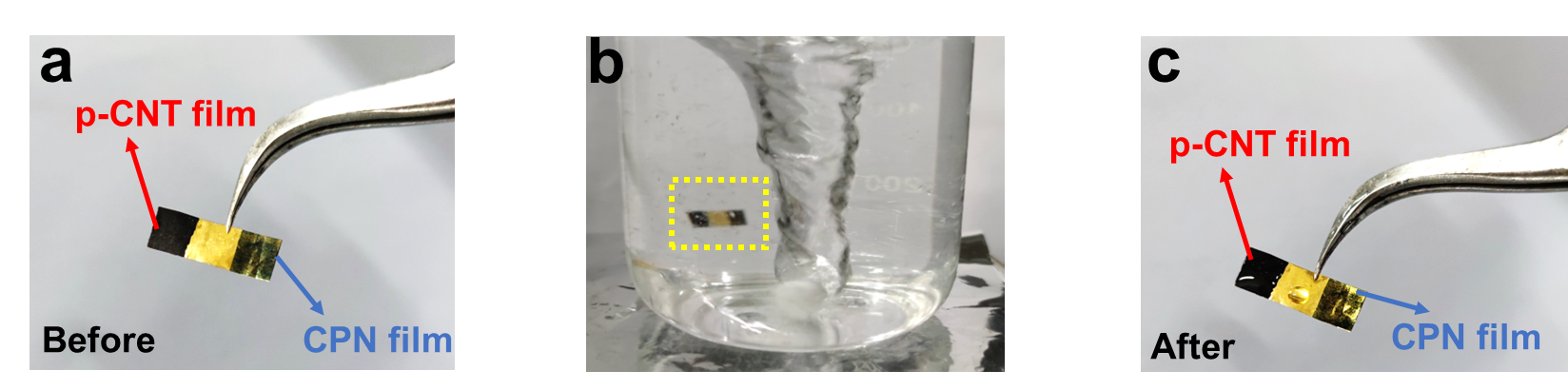


**Figure S19.** The excellent contact between electrode materials (CPN film and p-CNT film) and Au current collectors. Photographs of (a) as-prepared CPN film and p-CNT film on Au foil, (b) CPN film and p-CNT film on Au foil were being stirred in water, and (c) CPN film and p-CNT film on Au foils remain intact after stirring in water overnight.

In order to further test the close contact between the electrode materials and the Au current collector, the CPN film and p-CNT film on Au foil was immersed into water (Figure S19a). After stirring overnight (Figure S19b), the electrode materials are still intact and attached to the Au current collector (Figure S19c), indicating the intimate contact between electrode materials and Au foils.

**Table S1** Comparisons of the electrochemical performance parameters of various common aqueous and water-in-salt ECs for AC-line filtering.

| **Electrodes** | ***E*_A, 120_**  **[mF V^2^ cm^-2^]** | **−Phase angle [°]**  **at 120 Hz** | **τ_RC_**  **[ms]** | **τ_0_**  **[ms]** | ***C*_A, 120_**  **[mF cm^-2^]** | **Voltage**  **[V]** | **References** |
| --- | --- | --- | --- | --- | --- | --- | --- |
| **ACPEC-90^1^** | **0.55** | **84.0** | **0.14** | **0.26** | **0.49** | **1.5** | **This work** |
| **ACPEC-200^1^** | **1.29** | **83.3** | **0.15** | **0.46** | **1.15** | **1.5** | **This work** |
| **ACPEC-500^1^** | **1.72** | **77.6** | **0.30** | **0.82** | **1.53** | **1.5** | **This work** |
| **HPDEC-9^2^** | 0.54 | 84.0 | 0.14 | 0.56 | 1.09 | 1.0 | ^1^1 |
| **AT-PEDOT:PSS^3^** | 0.32 | 83.6 | 0.15 | 0.59 | 0.99 | 0.8 | ^2^2 |
| **AHECs^4^** | 0.44 | 82.0 | 0.18 | 0.46 | 0.27 | 1.8 | ^3^3 |
| **CNO-graphene^5^** | 0.07 | 82.2 | 0.17 | 0.22 | 0.15 | 1.0 | ^6^6 |
| **3-GPB-5^6^** | 0.41 | 85.9 | -- | 0.28 | 1.02 | 0.9 | ^7^7 |
| **4-GPB-5^6^** | 0.95 | 81.0 | -- | 0.99 | 2.34 | 0.9 | ^7^7 |
| **VOGNs^7^** | 0.04 | 82.0 | 0.20 | 0.07 | 0.09 | 1.0 | ^8^8 |
| **CB^8^** | 0.28 | 75.0 | 0.35 | 1.56 | 0.56 | 1.0 | ^9^9 |
| **ErGO^9^** | 0.09 | 84.0 | 1.35 | 0.24 | 0.28 | 0.8 | ^10^10 |
| **VOG^10^** | 0.15 | 82.0 | 0.21 | 0.25 | 0.36 | 0.9 | ^11^11 |
| **SWNT^11^** | 0.20 | 81.0 | 0.20 | 0.70 | 0.60 | 0.8 | ^12^12 |
| **NHG^12^** | 0.15 | 83.6 | 0.15 | 0.46 | 0.48 | 0.8 | ^13^13 |
| **EOG/CCP^13^** | 0.12 | 83.0 | - | 0.08 | 0.30 | 0.9 | ^14^14 |
| **EOG/CNF^14^** | 0.12 | 81.5 | - | 0.07 | 0.37 | 0.8 | ^15^15 |
| **GMF-200-5T^15^** | 0.10 | 82.3 | 0.32 | -- | 0.31 | 0.8 | ^16^16 |
| **MPHM^16^** | 0.18 | 79.1 | 0.29 | 0.71 | 0.56 | 0.8 | ^17^17 |
| **EG/PH1000^17^** | 0.01 | 75.0 | 0.57 | 1.50 | 0.03 | 1.0 | ^18^18 |
| **X,Y-BWC^18^** | 0.26 | 83.5 | 0.16 | 0.88 | 0.51 | 1.0 | ^19^10 |
| **VA-GMM_40_ ^19^** | 0.05 | 81.3 | 0.18 | 0.08 | 0.14 | 0.8 | ^20^20 |
| **CBC-20^20^** | 1.21 | 82.0 | 0.18 | 0.30 | 2.98 | 0.9 | ^21^21 |
| **KB-SWCNT^21^** | 2.38 | 78.7 | -- | 1.41 | 0.90 | 2.3 | ^22^22 |

^1^ACPEC-n = Aqueous hybrid electrochemical capacitor with continuous PEDOT nanomesh film as positive electrode and porous carbon nanotube film as negative electrode (n = 90, 200, 500 nm, the thickness of each electrode)

^2^HPDEC-9 = Electrochemical capacitor based on 250-nm-thick PEDOT:PSS films

^3^ AT-PEDOT:PSS = Acid treated PEDOT:PSS (AT-PEDOT:PSS);

^4^AHECs = Aqueous hybrid electrochemical capacitors (PEDOT||ErGO);

^5^CNO-graphene = Carbon nano-onion surrounded with graphene;

^6^GPB = Carbonized PB cubes with EOG deposition;

^7^VOGNs = Vertically oriented graphene nanosheets;

^8^CB = Carbon black;

^9^ErGO = Electrochemically reduced graphene oxide;

^10^VOG = Vertically oriented graphene;

^11^SWNT = Single walled multi-walled carbon nanotube film;

^12^NHG = Nitrogen-doped holey graphene;

^13^EOG/CCP= Edge-oriented multilayer graphene/thin-graphite in carbonized cellulous paper;

^14^EOG/CNF = 3D edge-oriented graphene (EOG) was grown encircling carbon nanofiber (CNF);

^15^GMF-200-5T = Graphene nanomesh film;

^16^MPHM = MXene/PEDOT:PSS hybrid materials;

^17^EG/PH1000 = Exfoliated graphene/PEDOT:PSS;

^18^X,Y-BWC = Carbon membrane from mechanically pressed X,Y-balsa wood;

^19^VA-GMM_40_= Vertically aligned graphene macroporous membrane;

^20^CBC-20 = Crosslinked carbon nanofiber aerogel with a thickness of 20 μm;

^21^KB-SWCNT = Ketjen black and single-walled carbon nanotubes.

**Notes:** Although two of the references (Ref.21 and Ref.7) show higher capacitance than ACPEC-200, other parameters including energy density, phase angle, and resistance-capacitance time constant (*τ*_RC_) are inferior to ACPEC-200 device of this work, indicating that our device still has comparable advantages in filtering applications. In addition, the reference of Ref.22 reported a wide voltage range of 2.3 V by using water-in-salt electrolyte, leading to a higher areal specific energy density. However, water-in-salt filtering capacitor can be regarded as a kind of special aqueous filtering capacitor. The high viscosity and low ionic conductivity of water-in-salt electrolyte usually cause water-in-salt filtering capacitors with lower frequency response, larger impedance, and smaller capacitance, comparing with common aqueous filtering capacitors (Supporting Information Table S1). These disadvantages in filtering applications would be amplified especially in high-voltage integration. Moreover, water-in-salt electrolyte is more toxic, corrosive and danger than common aqueous electrolyte. Under the comprehensive comparison, ACPEC device still shows great advantages in the development of water-based filtering capacitors.

**Table S2** The parameter comparison of n-ACPECs device presented in our work for AC line filtering. All parameters are measured at 120 Hz and room temperature of 25.8±0.1 °C, the resistance is equal to ESR of capacitors.

|  | ***C*_120_**  **[μF]** | **Volume of the device**  **[cm^3^]** | ***C*_v, device, 120_**  **[F cm^-3^]** | ***DF*_120_**  **[%]** | **Ripple current**  **[mA]** | **CV/volume_120_**  **[FV cm^-3^]** |
| --- | --- | --- | --- | --- | --- | --- |
| **ACPEC** | 287 | 7.56×10^-3^ | 3.8×10^-2^ | 10.7 | 0.3 | 5.7×10^-2^ |
| **7-ACPECs** | 40 | 5.9×10^-2^ | 6.8×10^-4^ | 10.6 | 0.2 | 6.8×10^-3^ |
| **67-ACPECs** | 4.6 | 5.9×10^-1^ | 7.8×10^-6^ | 15.8 | 0.2 | 7.8×10^-4^ |
| **670-ACPECs** | 0.5 | 5.9 | 8.5×10^-8^ | 25.3 | 67 | 8.5×10^-5^ |


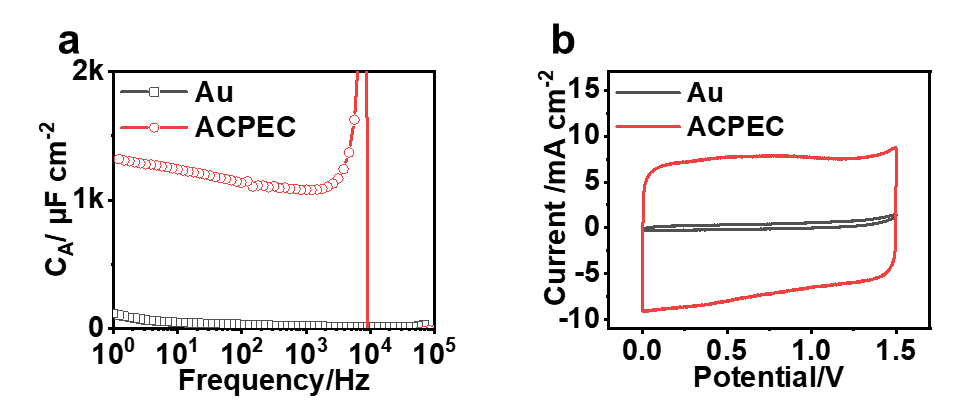


**Figure S20.** Comparison electrochemical performances of ACPEC and bare Au foils-based EC. (a) Plots of specific areal capacitance as function of frequency. (b) CV curves for the bare Au foils-based EC and ACPEC. Source data are provided as a Source Data file.


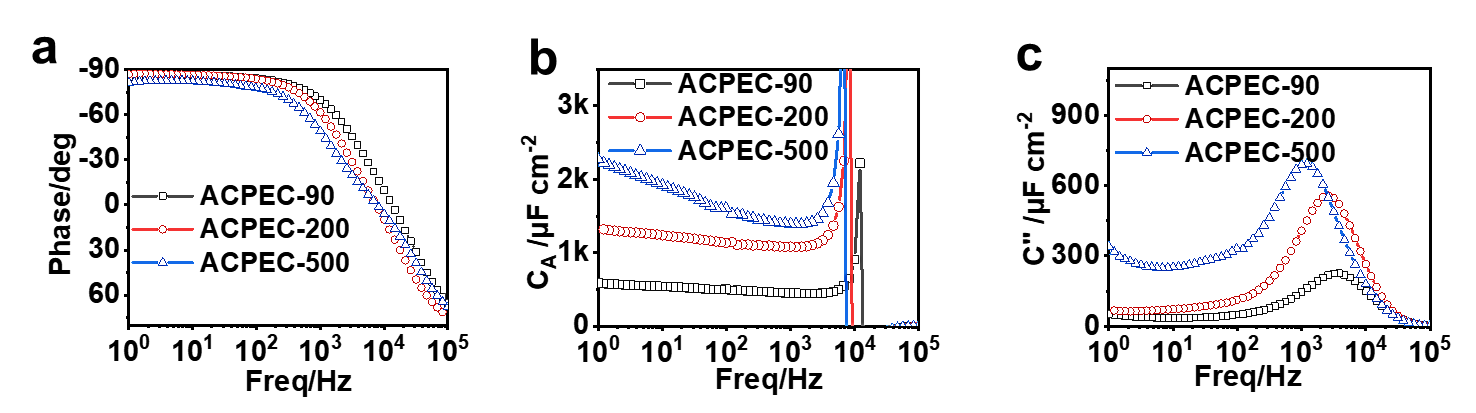


**Figure S21.** Electrochemical performances of ACPEC-90, ACPEC-200, and ACPEC-500. (a) Plots of phase angle versus frequency. (b) Plots of *C*_A_ versus frequency. (c) Plots of imaginary capacitance (*C*") versus frequency. Source data are provided as a Source Data file.

The *C*_A_ and frequency response performance of ACPEC-90, ACPEC-200, and ACPEC-500 strongly depend on the thickness of the film electrode (Table S1). At 120 Hz, the phase angles of ACPEC-90, ACPEC-200, and ACPEC-500 are −84°, −83.3°, and −77.6° (Figure S21a), while the corresponding areal specific capacitances at 120 Hz (*C*_A, 120_) are 0.49, 1.15, and 1.53 mF cm^-2^, respectively (Figure S21b). The areal specific energy densities at 120 Hz (*E*_A, 120_) of ACPEC-90, ACPEC-200, and ACPEC-500 also show an increasing trend and the values are 0.55, 1.29, and 1.72 mF V^2^ cm^-2^, respectively. The *τ*_RC_ values at 120 Hz increases from 0.14 ms to 0.3 ms with thickness increases from 90 to 500 nm. Similarly, the values of relaxation time constant (*τ*_0_) are turned out to be 0.26, 0.46, and 0.82 ms (Figure S21c). Overall, the electrochemical performance of ACPEC-n can be adjusted by controlling the thickness of the films. On the basis of comprehensive consideration of various electrochemical performance parameters, the thickness of the positive and negative electrodes used in this work is 200 nm.


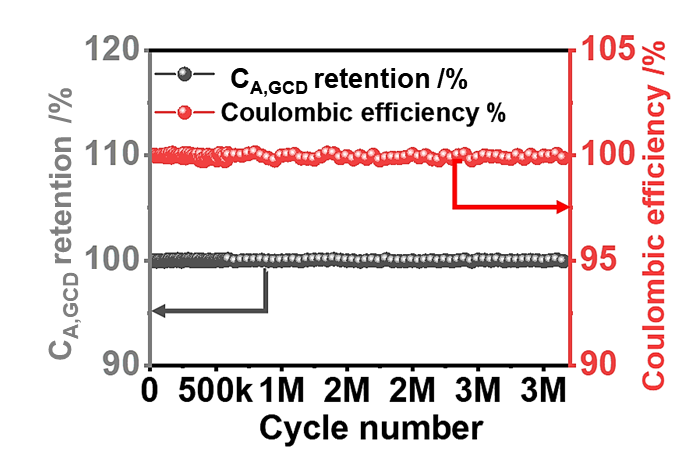


Figure S22. Cycling stability of ACPEC unit. The retention of areal specific capacitance and coulombic efficiency calculated from GCD curves at a discharging current density of 5 mA cm^-2^ for 8.5×10^6^ cycles. Source data are provided as a Source Data file.


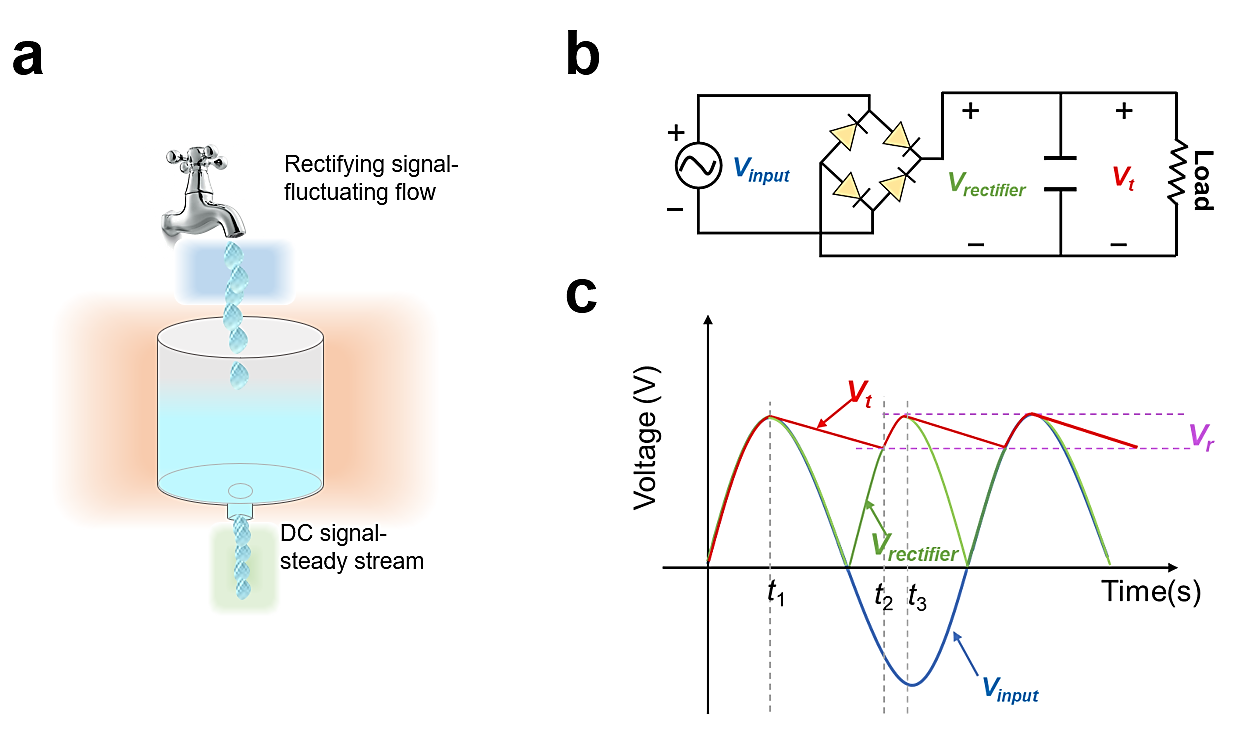


**Figure S23.** (a) Filtering capacitor functions like a reservoir that converts a fluctuating flow to a steady stream. (b, c) Conversion process of the sinusoidal AC waveform signal to DC signal, (b) is the filtering circuit, and (c) is the corresponding signals of (b).

In this filtering process, the capacitor is continuously charged and discharged just like a reservoir (Figure S23a). When the voltage rises above the voltage of the capacitor, the capacitor is charged. Then as the voltage drops, the capacitor discharges to stabilize the voltage. For a typical filtering circuit and corresponding process (Figure S23b,c), a sinusoidal AC signal is firstly converted into a signal of constant polarity after passing through a rectifier composed of four diodes. The output signal can be expressed as:

$V_{input}=|V_{0}\sin(\omega t)|$ (1)

where $V_{input}$ is the voltage signal of inputting to rectifier, $V_{0}$ is the maximum value of input AC voltage, $\omega$ is angular frequency, $t$ is time. Subsequently, the rectified signal ($V_{rectifier}$) is further smoothed by the filtering capacitor to output a straight line with ripple voltage ($V_{r}$). Therefore, the$V_{r}$ mainly comes from the voltage drop caused by the discharge of the capacitor. Furthermore, transient voltage ($V_{t}$) of the capacitor could be described as:

$V_{t}=V_{0}e^{\frac{-t}{RC}}$ (2)

And $V_{r}$ could be calculated according to the equation (3)^23,24^.

$V_{r}=V_{0}-V_{t}=V_{0}(1-e^{\frac{-t}{RC}})$ (3)

where *C* is the capacitance of the filtering capacitor. *R* is the total resistance of load resistor and capacitor.

According to the equation (3), it can be concluded that $V_{r}$ is positively correlated with t/RC under a typical series-RC circuit model^23^. Notably, the ripple voltage will attenuate as the capacitance increases. Therefore, the large areal specific capacitance and ideal double-layer capacitive behavior of ACPEC ensure its excellent filtering ability.

The ripple current can be calculated as^24^:

$I_{r}=V_{r}/R$ (4)

The dissipation power ($P_{d}$) of capacitor can be calculated as^25^:

$P_{d}=ESR\times I_{r}^{2}$ (5)

For the typical 5% of ripple limitation in circuit design^25^, a 1 kΩ resistor is connected into the circuit under 25 ℃ to practically evaluate the ripple voltage of ACPEC and AEC (220 μF/16 V, CHONGX, China) at 120 Hz. As shown in Figure S24, the ripple voltage of ACPEC is 23 mV, twice smaller than AEC (220 μF/16 V, CHONGX, China) of 50 mV, indicating the better filtering performance of ACPEC.


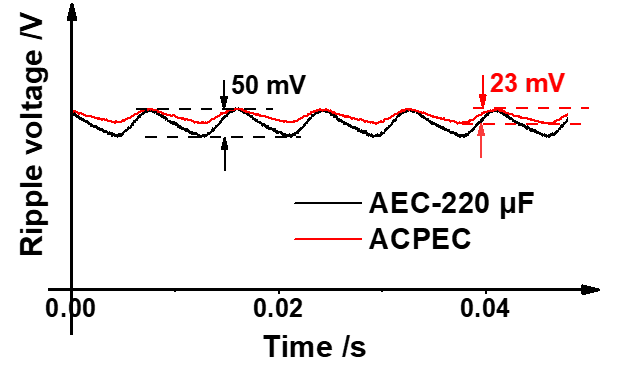


**Figure S24.** The ripple voltage of ACPEC unit and AEC (220 μF/16 V, CHONGX, China) with the load resistance of 1 kΩ under room temperature of 25 ℃. Source data are provided as a Source Data file.

**
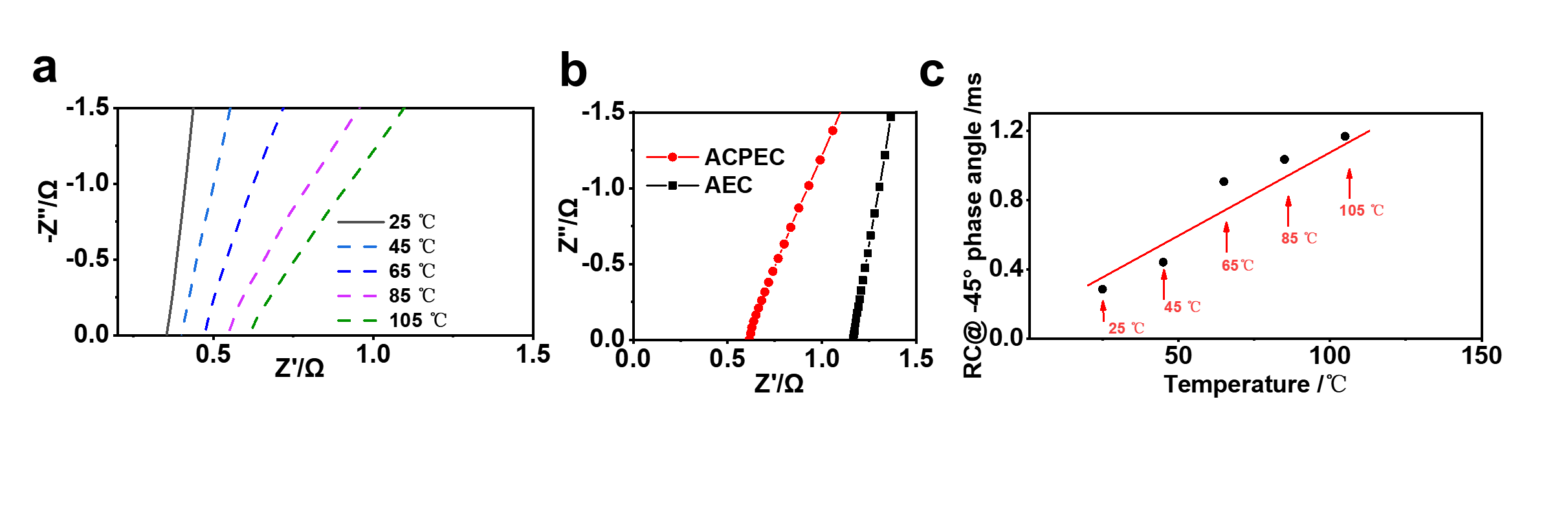
**

**Figure S25**. (a) Complex plane plots of the impedance of ACPEC unit measured at five different temperatures. (b) The Nyquist plots of ACPEC unit and AEC (220 μF/16 V, CHONGX, China) under temperature of 105 °C. (c) Plot of the ACPEC unit response time versus temperature. In comparison, commercial EDLCs have an RC product (in the 0.3 s to 2 s range). Source data are provided as a Source Data file.

The high-temperature performance of ACPEC unit at temperatures up to 105 °C was evaluated. Figure S25a shows a complex-plane plot of the impedance at five different temperatures. We find that there are no any distributed charge storage behaviors under all of the temperatures. The ESR increases with increasing temperature, but is still less than that of AEC (220 μF/16 V, CHONGX, China, Figure S25b) under temperature of 105 °C. The characteristic response time of ACPEC, i.e. its RC-time-constant calculated at a frequency where the phase angle is –45°, is ~0.3 ms at room temperature and increases to ~1.2 ms at 105°C (Figure S25c), which is smaller than commercial EDLCs with an RC time product (typically in the 0.3 s to 2 s range).


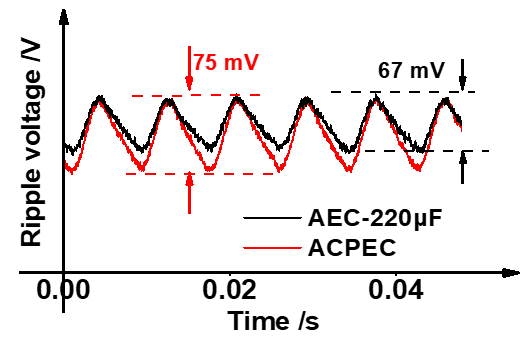


**Figure S26**. The maximum ripple voltage of ACPEC and AEC (220 μF/16 V, CHONGX, China) with the load resistance of 1 kΩ under temperature of 105 °C. Source data are provided as a Source Data file.

In Figure S26, under the highest allowable operating temperature of 105 °C and load of 1 kΩ, the ripple voltage of ACPEC unit is measured as 75 mV, and the maximum ripple current is calculated to be 75 μA, which is higher than that of AEC (67 μA, 220 μF/16 V, CHONGX, China). Meanwhile, the dissipation power of ACPEC (3.5 nW, ESR = 0.63 Ω) is smaller than that of AEC (5.4 nW, ESR=1.2 Ω), indicating the less power consumption and higher maximum allowed ripple current of ACPEC.


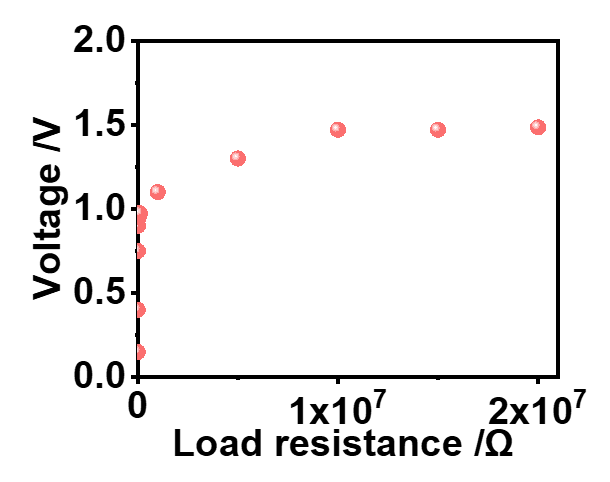


**Figure S27**. The output voltage of ACPEC versus the load resistance connected in parallel. Source data are provided as a Source Data file.

In order to optimize the output voltage of ACPEC unit, the loads with different resistance values are connected in circuit. As shown in Figure S27, the output voltage increases with the increasing value of load resistance, and maintains stable when the load resistance goes up to 10 MΩ. Therefore, we choose the 10 MΩ connected in circuit during the filtering test in the following part.


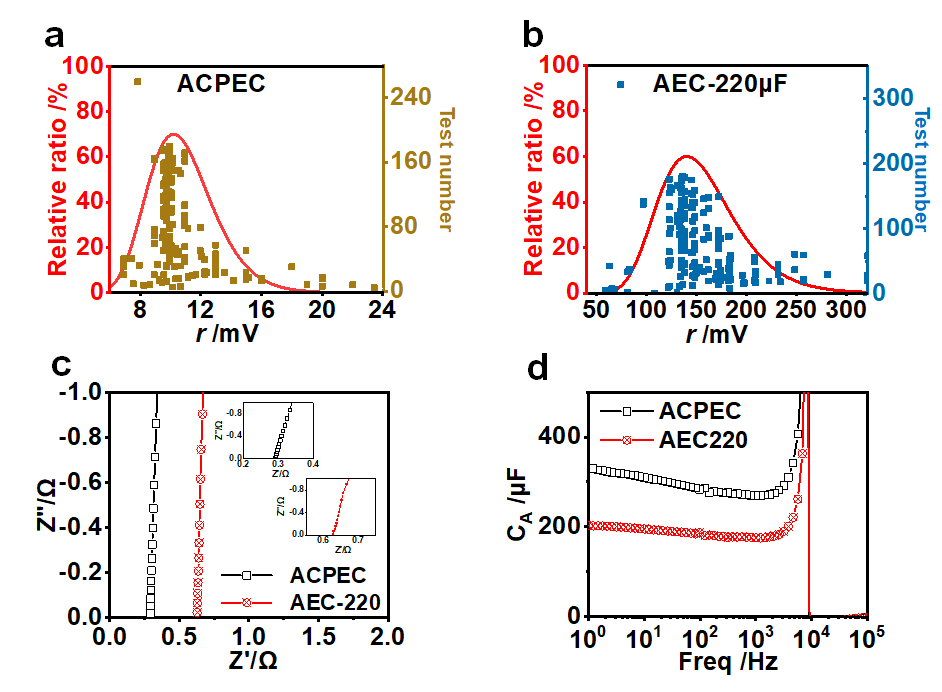


**Figure S28**. Comparison of the value of fluctuation for ACPEC (287 μF) and AEC (220 μF/16 V, CHONGX, China). The plots of normal distributions of *r* for (a) ACPEC (287 μF) and (b) AEC (220 μF/16 V, CHONGX, China). Source data are provided as a Source Data file.

As shown in Figure S28, the normal distributions of fluctuation for ACPEC (287 μF) and AEC (220 μF/16 V, CHONGX, China) were obtained by 180 parallel tests by using oscilloscope (RTB2002, Rohde & Schwarz, Germany), respectively. As can be seen, the fluctuation of ACPEC is mainly around 9~12 mV, ~13 times smaller than that of AEC (120~180 mV, 220 μF/16 V, CHONGX, China).


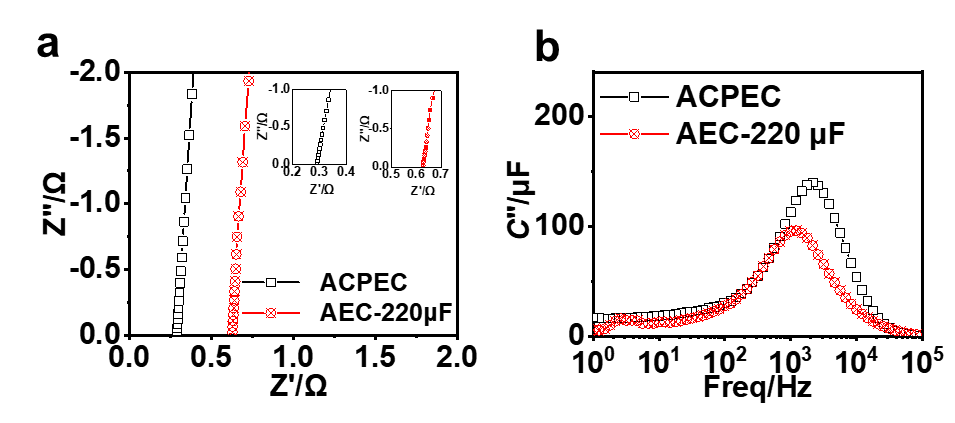


**Figure S29.** The electrochemical performances of ACPEC (287 μF) and AEC (220 μF/16 V, CHONGX, China). (a) Nyquist plots. Insets: the magnified Nyquist plots at high frequency. (b) Plots of *C*" versus frequency of ACPEC (287 μF) and AEC (220 μF/16 V, CHONGX, China). Source data are provided as a Source Data file.

From the Figure S29a, the ESR of the single ACPEC (287 μF) unit is 0.28 Ω, 2.3 times smaller than that of AEC (0.63 Ω, 220 μF/16 V, CHONGX, China). And the relaxation time constant (*τ*_0_) (Figure S29b) of ACPEC (0.46 ms) is also less than AEC (1 ms, 220 μF/16 V, CHONGX, China). Both of them can reflect that faster frequency response of ACPEC can benefit to output smoother DC signal, which is consistent with the results of oscilloscope (RTB2002, Rohde & Schwarz, Germany) test.


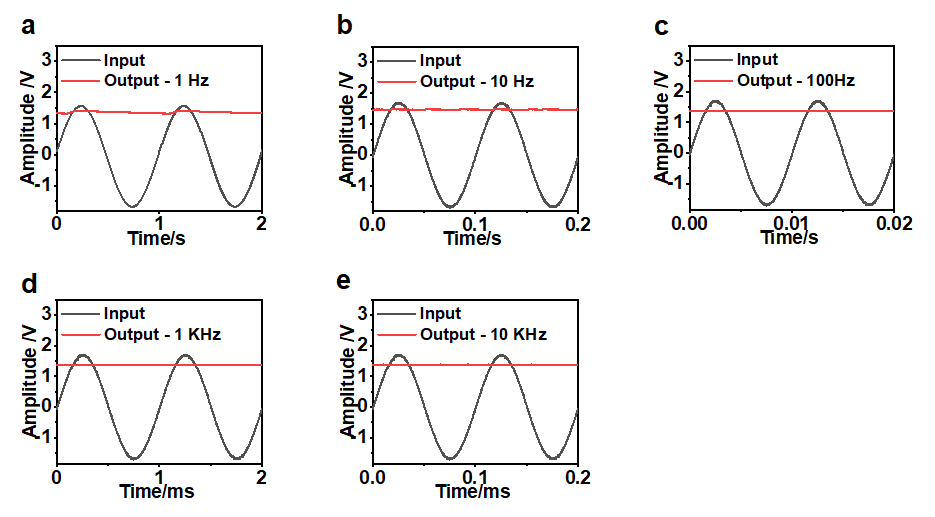


**Figure S30.** (a-f) The filtering performance of ACPEC at different frequencies. Source data are provided as a Source Data file.


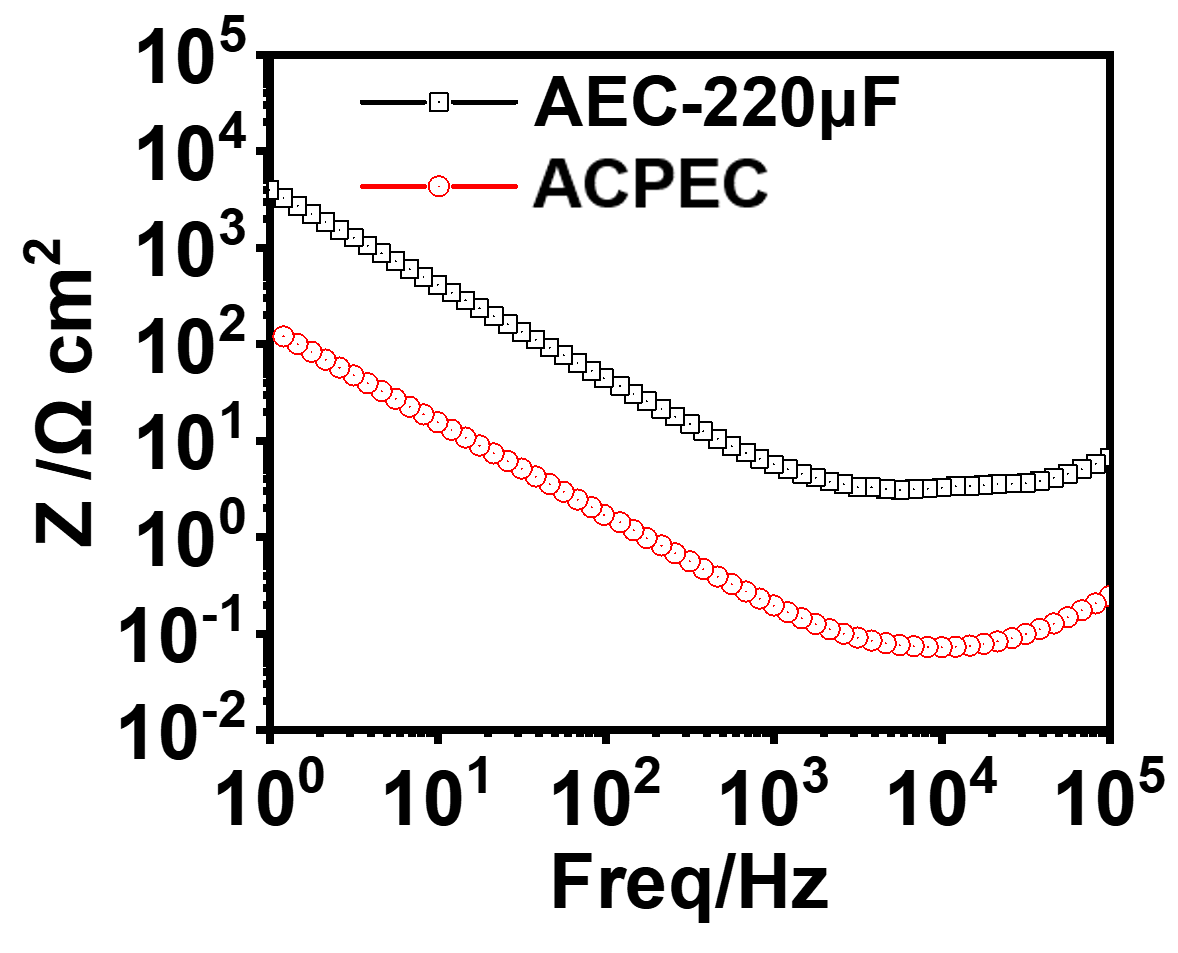


**Figure S31.** Plots of total impedance of AEC (220 μF/16 V, CHONGX, China) and ACPEC at the frequency ranges of 1 Hz to 100,000 Hz. Source data are provided as a Source Data file.

In general, for most capacitors, the typical equivalent circuit can be described as an RCL circuit (a resistor (R), a capacitor (C), and an inductor in series (L)). Therefore, total impedance (*Z*) has the following equation:

$$Z=\sqrt{ESR^{2}+\left(\frac{1}{2\pi fC}-2\pi fL\right)^{2}}$$

where *f* is the frequency, *C* is the capacitance, and *L* is the inductance.

In supplementary Figure S31, the *Z* of AEC and ACPEC within the frequency range from 1 Hz to 100,000 Hz are presented. For all the frequency ranges, the *Z* of ACPEC is smaller than that of AEC. The frequency where $1/2\pi fC=2\pi fL$is known as self-resonant frequency (SRF). It is also the lowest point in the curve of *Z* versus frequency. For the two-terminal electrical component, the SRF is also the separation determining that the component behaves like a capacitor or an inductor. In our ACPEC, the SRF is approximately 10 kHz, and below the 10 kHz, they can be used as the filtering capacitor. Therefore, the smaller *Z* can be attributed to the lower ESR (0.28 Ω) and larger capacitance of ACPEC (287 μF) within the workable frequency range.


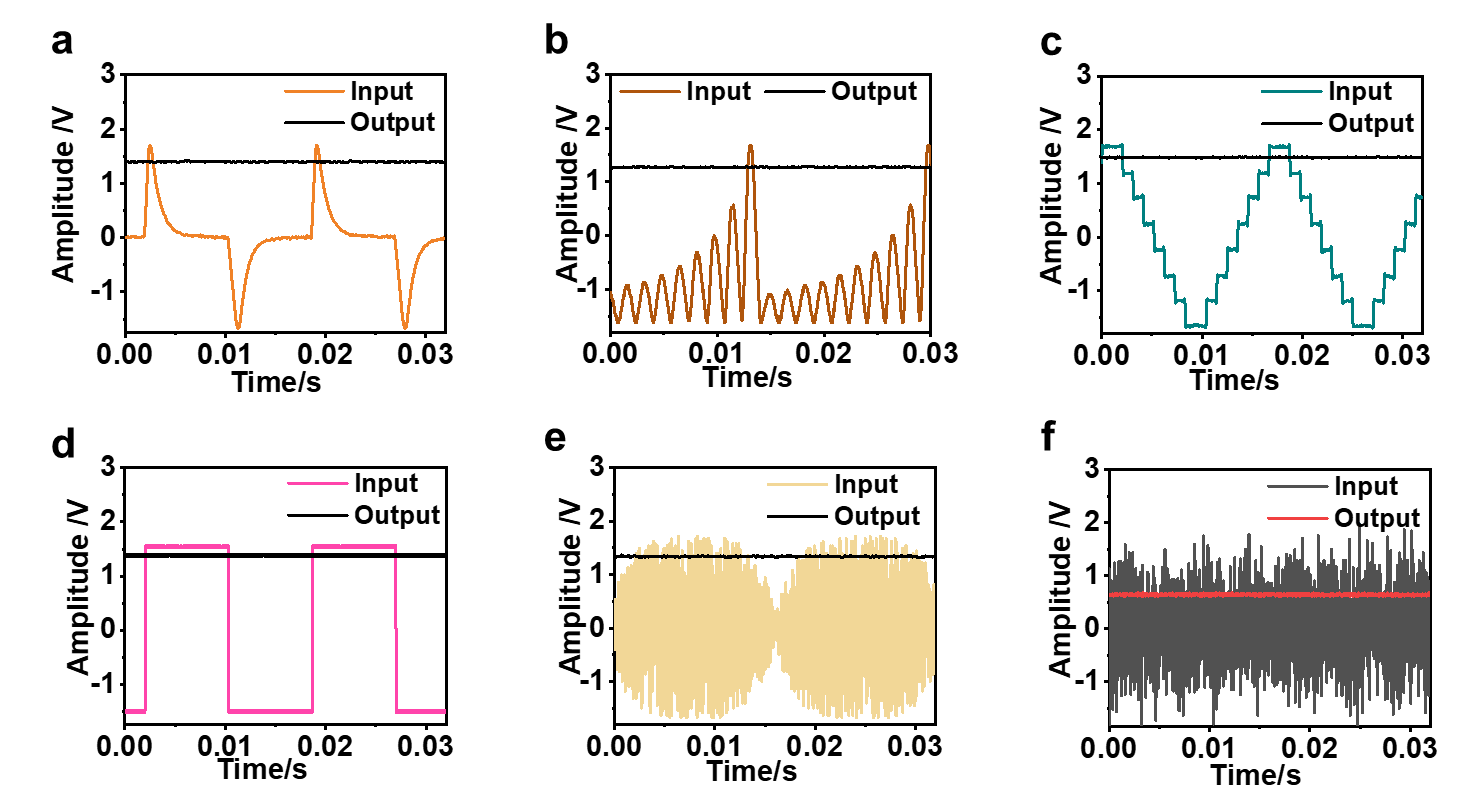


**Figure S32.** The filtering performance of an ACPEC unit for arbitrary waveforms. The input AC waveform signals (3.2 V_peak-peak_) are (a) CAP-vol, (b) Dempedosc, (c) ramp, (d) square, (e) circle and (f) noise waveforms. The frequency from (a) to (f) is 60 Hz. Source data are provided as a Source Data file.


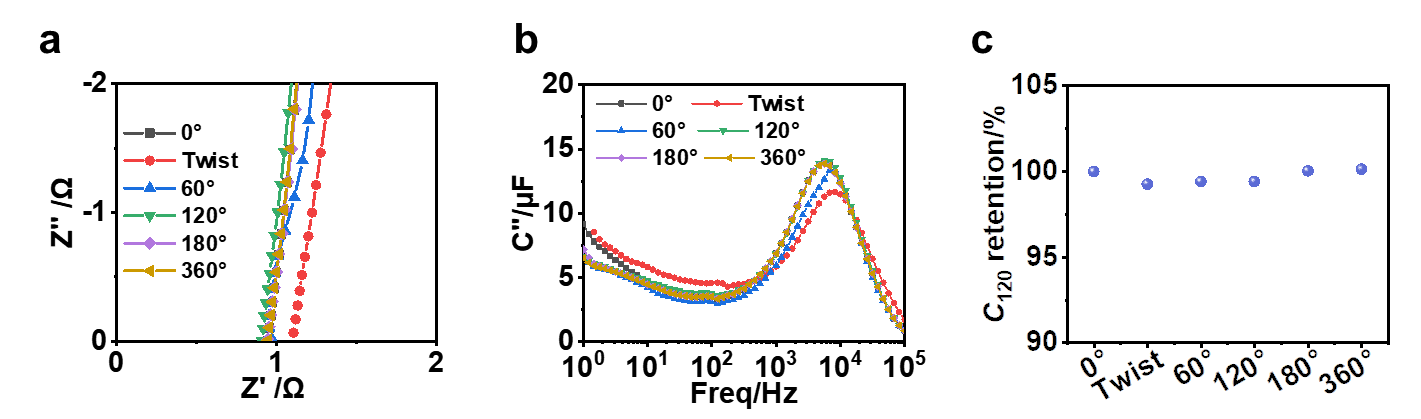


**Figure S33.** (a) Nyquist plots, (b) the plots of the imaginary part of *C*" versus frequency, and (c) the capacitance retention of 7-ACPECs at different deformation states. Source data are provided as a Source Data file.


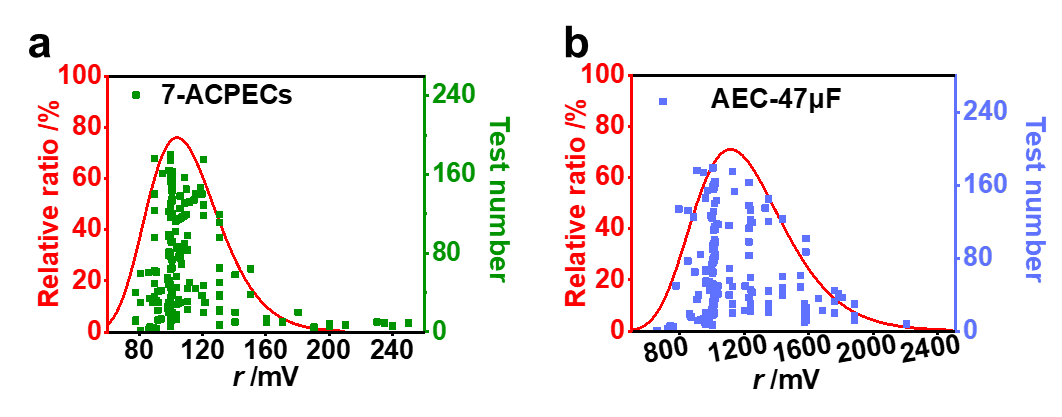


**Figure S34.** Comparison of the value of fluctuation for 7-ACPECs (40 μF/10 V) at initial state of 0° and AEC (47 μF/16 V, CHONGX, China). The plots of normal distributions of *r* for (a) 7-ACPECs (40 μF/10 V) and (b) AEC (47 μF/16 V, CHONGX, China). Source data are provided as a Source Data file.

As shown in Figure S34, the normal distributions of fluctuation for 7-ACPECs (40 μF/10 V) and AEC (47 μF/16 V, CHONGX, China) were obtained by 180 parallel tests with oscilloscope (RTB2002, Rohde & Schwarz, Germany), respectively. As can be seen, the *r* of 7-ACPECs is mainly around 100 mV, ~10 times lower than that of AEC (900 mV ~1200 mV, 47 μF/16 V, CHONGX, China).


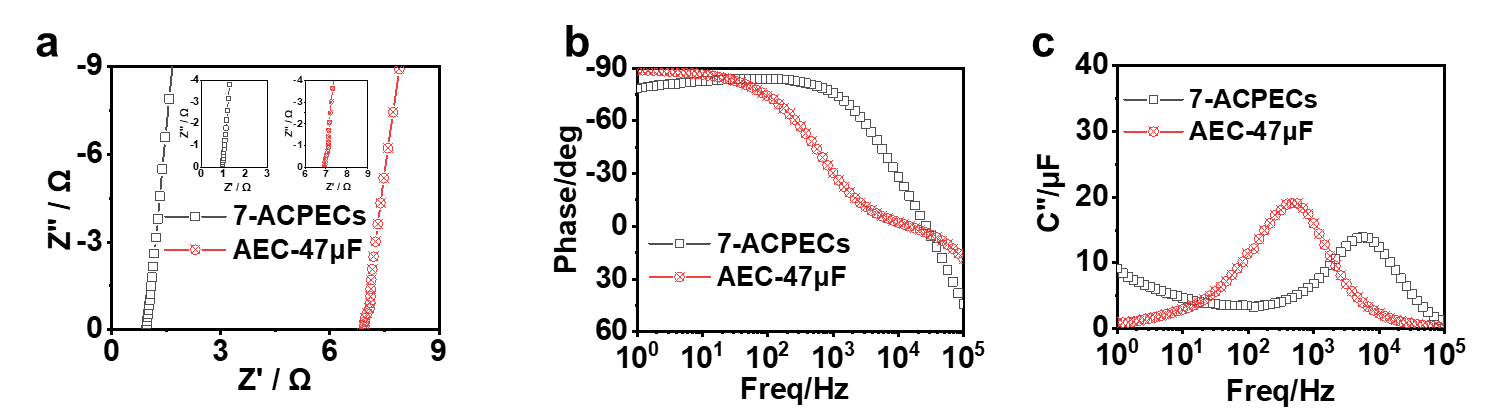


**Figure S35**. Comparison of the electrochemical performance of 7-ACPECs (40 μF/10 V) and AEC (47μF/16 V, CHONGX, China). (a) Nyquist plots. (b) Bode plots. (c) Plots of the imaginary part of *C*" versus frequency. Source data are provided as a Source Data file.

As shown in Figure S35, comparing with the 7-ACPECs, the Nyquist plot of the AEC (47 μF/16 V, CHONGX, China) exhibits an ESR of 7.2 Ω, about 7.2 times larger than 7-ACPECs of 1 Ω (Figure S35a). And there is a knee point and the obviously followed 45° region in high frequency region, implying the obvious porous electrode behavior in the electrodes and slower frequency response performance of AEC (47 μF/16 V, CHONGX, China)^26,27^. Except that, the reasons why the fluctuation of 7-ACPECs (40 μF/10 V) is 10 times lower than that of AEC (47 μF/16 V, CHONGX, China) may be attributed to the following aspects:

On the one hand, the phase angle (Figure S35b) and relaxation time constant (*τ*_0_) (Figure S35c) of AEC (47 μF/16 V, CHONGX, China) are inferior than 7-ACPECs (40 μF/10 V), which indicates the slower frequency response of AEC and may induce a large fluctuation. On the other hand, comparing with the crimped structured and low conductive aluminum foil current collectors of AEC, the 7-ACPECs is a simple two-electrode mode of sandwich structure and seamlessly connected with Au foil current collectors, which is more benefit to heat dispersion directly and improving the filtering performance of 7-ACPECs.


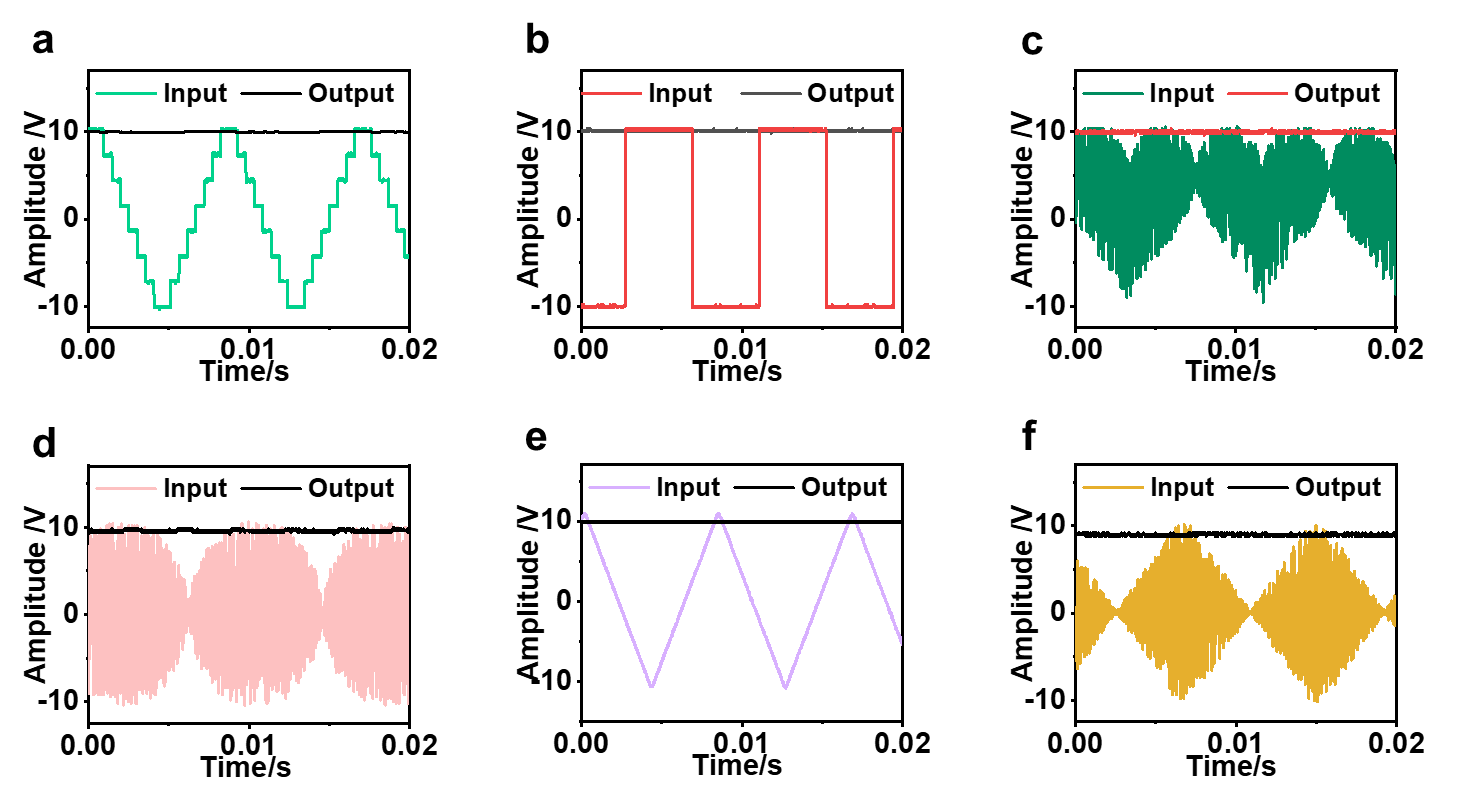


**Figure S36.** Filtering performances of 7-ACPECs at bending angle of 0°. The input AC waveform (21 V_peak-peak_) signals are (a) stair, (b) square, (c) heart, (d) circle, (e) triangle, (f) diamond waveforms. The frequency in (a) to (f) is 60 Hz. Source data are provided as a Source Data file.

**
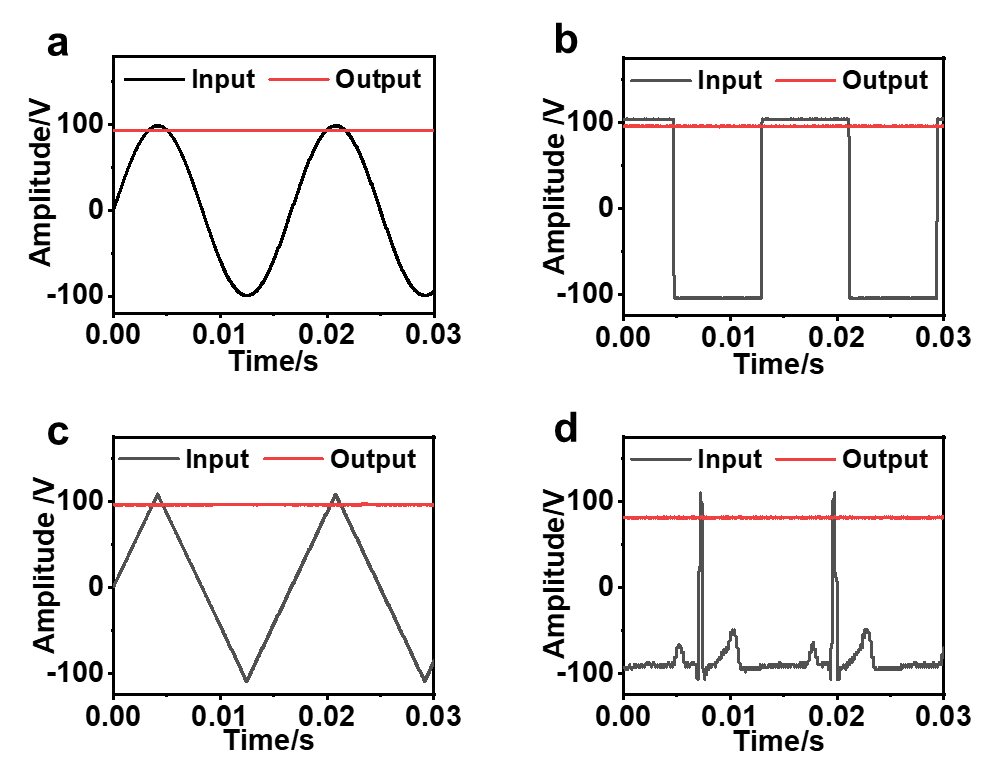
**

**Figure S37.** Filtering performances of 67-ACPECs. The input AC waveform signals (200 V_peak-peak_) are (a) sin, (b) square, (c) triangle, (d) electrocardiogram waveforms. The frequency is 60 Hz. Source data are provided as a Source Data file.


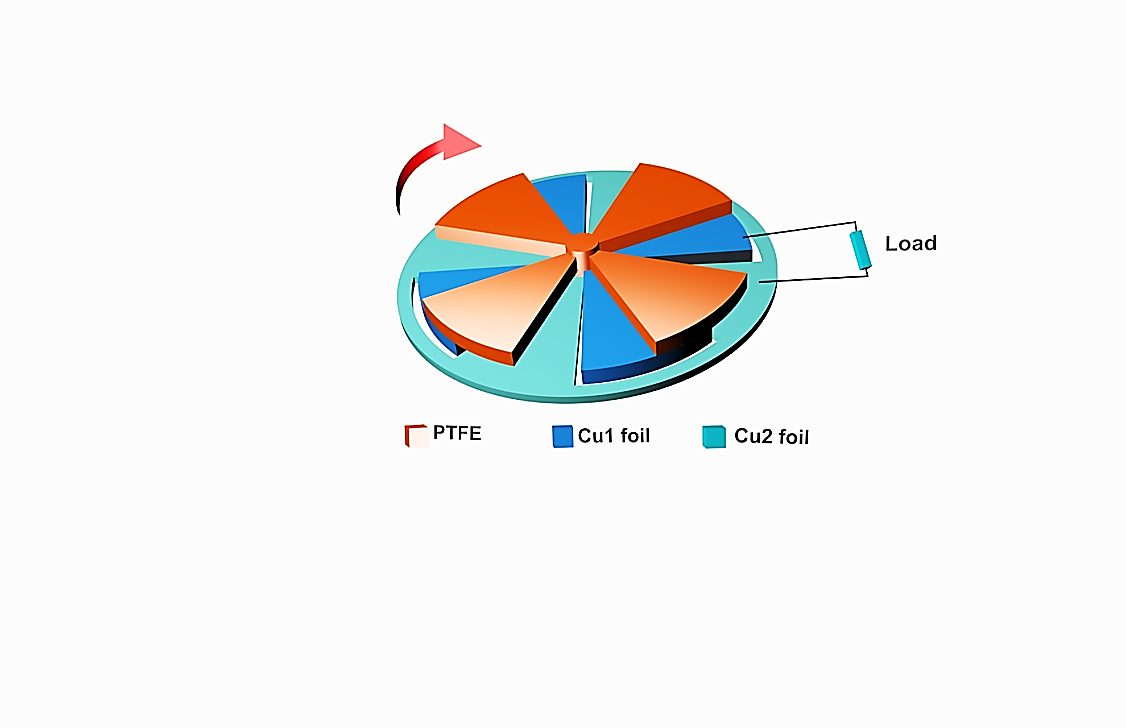


**Figure S38.** The schematic of the basic structure of the RD-TENG composed of the PTFE layer and the stationary Cu layer. The bottom inset is the figure legend.


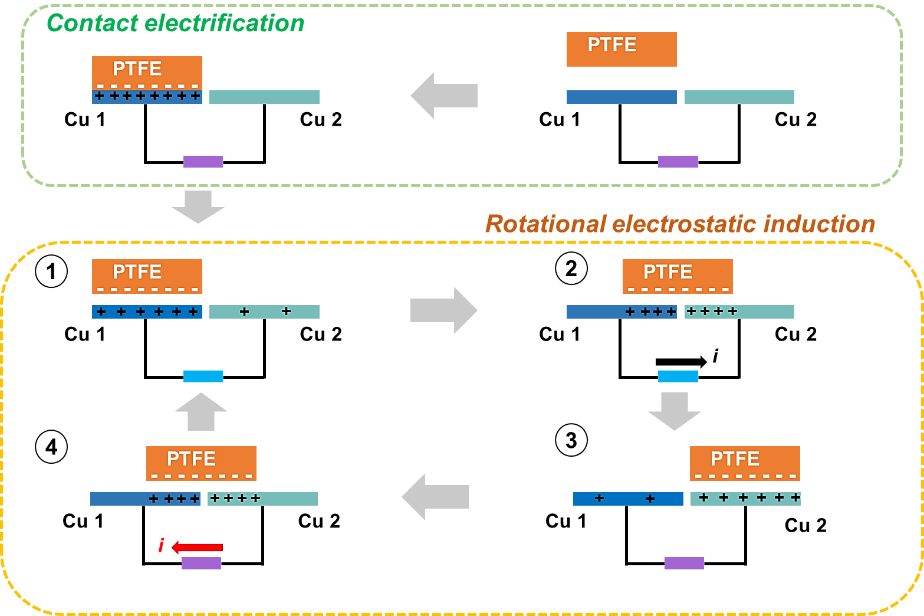


**Figure S39.** Schematic illustrations the working principle of the RD-TENG in a full cyclic motion of the rotational disk.


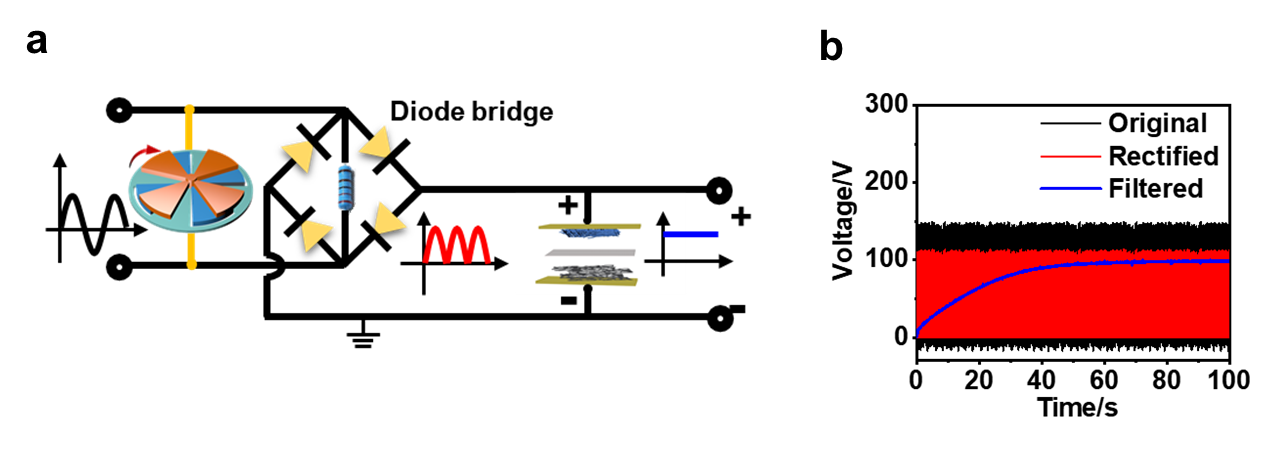


**Figure S40.** Integrating 67-ACPECs into the filtering circuit with RD-TENG as the power source. (a) Schematic: demonstration of this circuit for integrating 67-ACPECs with RD-TENG to obtain smooth output voltage signal. (b) The output voltage signals across original RD-TENG, rectifier and 67-ACPECs, respectively. Source data are provided as a Source Data file.


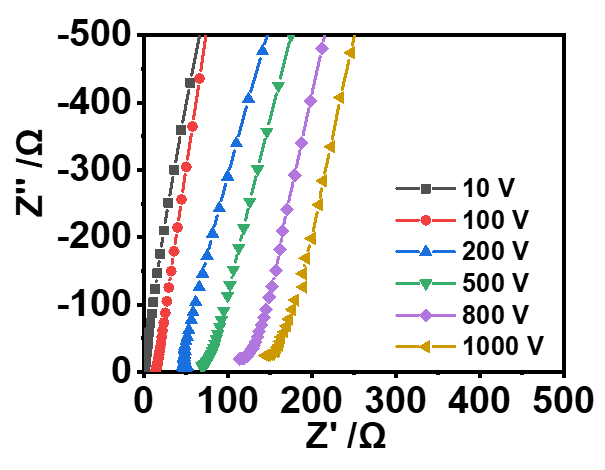


**Figure S41.** The Nyquist plots of the integrated filtering capacitors with various number of ACPEC units connected in series. Source data are provided as a Source Data file.

**Table S3** The highest voltage of integrated capacitors for AC line filtering.

| **Samples** | **Integrated highest voltage (V)** | **references** |
| --- | --- | --- |
| **ACPECs** | **1,000** | **This work** |
| HPDEC-9 | 12 | ^1^1 |
| CNO-graphene | 7.5 | ^6^6 |
| AHECs | 200 | ^3^3 |
| MXene | 60 | ^28^26 |

ACPECs = Aqueous hybrid electrochemical capacitors with continuous PEDOT nanomesh film as positive electrode and porous carbon nanotube film as negative electrode.

HPDEC-9 = Electrochemical capacitor based on 250-nm-thick PEDOT:PSS films

CNO-graphene = Carbon nano-onion surrounded with graphene;

AHECs = Aqueous hybrid electrochemical capacitors (PEDOT||ErGO);

MXene = MXene-based micro-supercapacitor


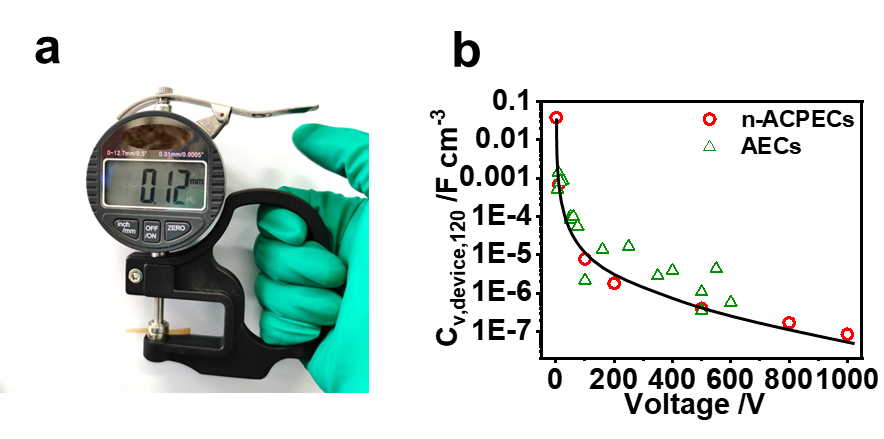


**Figure S42**. (a) The photo of the total thickness test of the ACPEC unit. (b) The plot of the areal volume capacitance (*C*_v, device, 120_) of n-ACPECs device versus output voltage of n-ACPECs presented in our work, along with the corresponding values of commercial AECs. The parameters of AECs are collected from the previous literatures^3,29^ and local store. Source data are provided as a Source Data file.

The comparison of *C*_v, device, 120_ between the n-ACPECs device and commercial AECs is exhibited in Figure S42. For each of the ACPEC unit, the output voltage is approximately 1.5 V, the areal specific capacitance (*C*_120_) is 287 μF at 120 Hz, and the corresponding *C*_v, device, 120_ is calculated to be 3.8×10^-2^ F cm^-3^. The total thickness including collectors, electrodes, separator, and uncompacted gaps is 120 μm (Figure S42a). The plots of *C*_v, device, 120_ versus output voltages for integrated n-ACPECs (red circle) and the fitted curve (black line), as well as the corresponding values of commercial AECs (green triangle), are presented in Figure S42b.


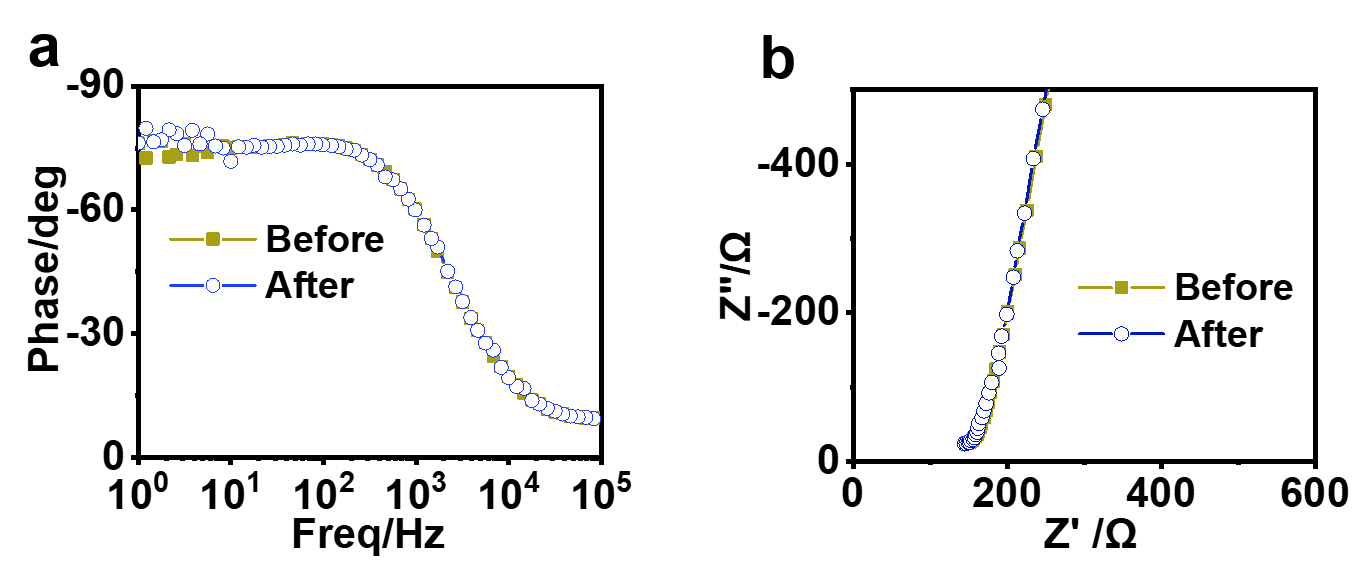


**Figure S43.** Comparison of the electrochemical performance of 670-ACPECs before and after charging/discharging. (a) Bode plots and (b) Nyquist plots of 670-ACPECs before and after charging/discharging. Source data are provided as a Source Data file.


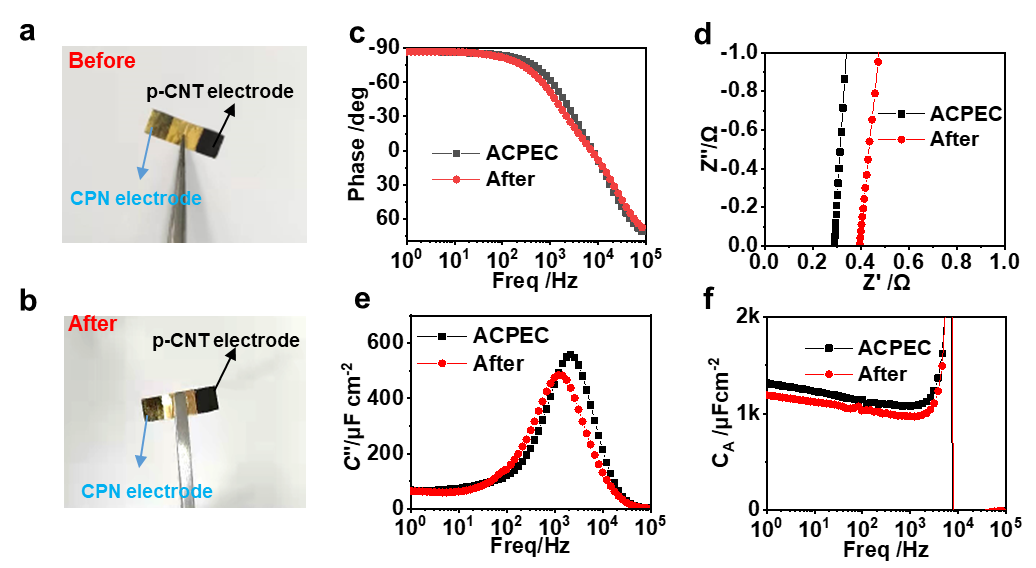


**Figure S44.** The stability demonstration of electrodes in 670-ACPECs. The photos of (a,b) positive CPN film and negative p-CNT film of disassembled ACPEC units from 670-ACPECs after stability test for 50 days. The comparison of electrochemical performance for initial ACPEC unit and reassembled hybrid capacitor unit after stability test for 50 days: (c) Plots of phase angle versus frequency, (d) the Nyquist plots, (e) plots of *C*" versus frequency, and (f) *C*_A_ of versus frequency. Source data are provided as a Source Data file.

As shown in Figure S44a,b, even after stability test for 50 days, the positive CPN and negative p-CNT films disassembled from one of ACPEC units in 670-ACPECs were still intact. To further demonstrate, these two electrodes were reassembled into a hybrid capacitor with only a slight decrease in electrochemical impedance performance (phase angle of **−**81.0° at 120 Hz, ESR of 0.4 Ω, *τ*_RC_ of 0.17 ms at 120 Hz, *τ*_0_ of 0.83 ms, and *C*_A_ of 1.03 mF cm^-2^ at 120 Hz, Figure S44c**−**f), comparing to those of pristine ACPEC unit (phase angle of **−**83.3°, ESR of 0.28 Ω, *τ*_RC_ of 0.15 ms at 120 Hz, *τ*_0_ of 0.46 ms and *C*_A_ of 1.15 mF cm^-2^ at 120 Hz).


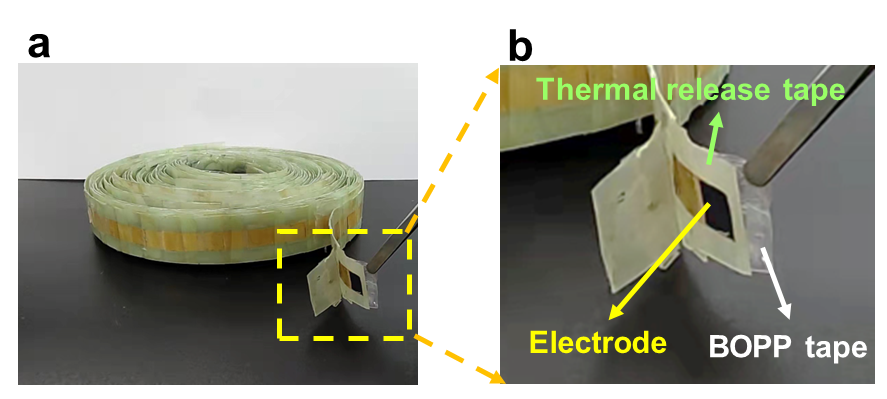


**Figure S45.** (a) The photos of 670-ACPECs fitted first with a thermal release tape, and then wrapped again with BOPP tape. (b) Magnified photo of that framed with yellow dotted line in (a).

**Supplementary References**

1. Zhou, L. *et al.* Continuous nanomesh PEDOT:PSS film: towards aqueous AC line filtering capacitor with ultrahigh energy density. *Chem. Eng. J.* **430**, 133012 (2021).
2. Miao, Zhang. *et al.* An ultrahigh-rate electrochemical capacitor based on solution-processed highly conductive PEDOT:PSS films for AC line-filtering. *Energy Environ. Sci*. **9**, 2005–2010 (2016).
3. Mingmao, W. *et al.* Arbitrary waveform AC line filtering applicable to hundreds of volts based on aqueous electrochemical capacitors. *Nat. Commun.* **10**, 2855 (2019).
4. Bo, Y. *et al.* High temperature rearrangement of disordered nanoporous carbon at the interface with single wall carbon nanotubes. *Carbon* **47**, 2303–2309 (2009).
5. Pc., E., Ra., J. & Jm., H. Vibrational modes of carbon nanotubes-spectroscopy and theory. *Carbon* **33**, 959–972 (1995).
6. Chenguang, Z., Haozhe, D., Ke, M. & Zhihao, Y. Ultrahigh-rate supercapacitor based on carbon nano‐onion/graphene hybrid structure toward compact alternating current filter. *Adv. Energy Mater.* **10**, 2002132 (2020).
7. Wenyue, L., Sakibul, A., Guangzhen, D. & Zhaoyang, F. Prussian blue based vertical graphene 3D structures for high frequency electrochemical capacitors. *Energy Storage Mater.* **32**, 30–36 (2020).
8. Miller, J. R., Outlaw, R. A. & Holloway, R. C. Graphene double-layer capacitor with ac line-filtering performance. *Science* **329**, 1637–1639 (2010).
9. Kossyrev, P. *et al.* Carbon black supercapacitors employing thin electrodes - ScienceDirect. *J. Power Sources* **201**, 347–352 (2012).
10. Kaixuan S., Yiqing S., Chun L., Wenjing Y. & Gaoquan S. Ultrahigh-rate supercapacitors based on eletrochemically reduced graphene oxide for ac line-filtering. *Sci. Rep.* **2**, 247 (2012).
11. Guofeng, R., Xuan, P., Stephen, B. & Zhaoyang, F. Kilohertz ultrafast electrochemical supercapacitors based on perpendicularly-oriented graphene grown inside of nickel foam. *Carbon* **71**, 94–101 (2014).
12. Rangom, Y., Tang, X. S. & Nazar, L. F. Carbon nanotube-based supercapacitors with excellent ac line filtering and rate capability via improved interfacial impedance. *ACS Nano* **9**, 7248–7255 (2015).
13. Zhou, Q., Zhang, M., Chen, J., Hong, J. D. & Shi, G. Nitrogen-doped holey graphene film-based ultrafast electrochemical capacitors. *ACS Appl. Mater. Inter.* **8**, 20741–20747 (2016).
14. Guofeng, R., Shiqi, L., Zhao-Xia, F., Md Nadim Ferdous, H. & Zhaoyang, F. Ultrahigh-rate supercapacitors with large capacitance based on edge oriented graphene coated carbonized cellulous paper as flexible freestanding electrodes. *J. Power Sources* **325**, 152–160 (2016).
15. Islam, N., Warzywoda, J. & Fan, Z. Edge-oriented graphene on carbon nanofiber for high-frequency supercapacitors. *Nano-Micro Lett.* **10**, 9 (2018).
16. Zheye, Z. *et al.* Scalable fabrication of ultrathin free-standing graphene nanomesh films for fexible ultrafast electrochemical capacitors with AC line-filtering performance. *Nano Energy* **50**, 182–191 (2018).
17. Gund, G. S. *et al.* MXene / polymer hybrid materials for flexible AC-filtering electrochemical capacitors. *Joule* **3**, 164–176 (2019).
18. Zhong-Shuai, W., Zhaoyang, L., Khaled, P., Xinliang, F. & Klaus, M. Ultrathin printable graphene supercapacitors with AC line‐filtering performance. *Adv. Mater.* **27**, 3669–3675 (2015).
19. Miao, Z. *et al.* From wood to thin porous carbon membrane: ancient materials for modern ultrafast electrochemical capacitors in alternating current line filtering. *Energy Storage Mater.* **35**, 327–333 (2020).
20. Miller, J. R., Outlaw, R. A. & Holloway, B. C. Graphene electric double layer capacitor with ultra-high-power performance. *Electrochim. Acta* **56**, 10443–10449 (2011).
21. Islam, N. *et al.* High-frequency electrochemical capacitors based on plasma pyrolyzed bacterial cellulose aerogel for current ripple filtering and pulse energy storage. *Nano Energy* **40**, 107–114 (2017).
22. Jinwoo, P., Junyoung, L. & Woong, K. Water-in-salt electrolyte enables ultrafast supercapacitors for AC line filtering. *ACS Energy Lett.* **6**, 769–777 (2021).
23. Dorf, R. C. The engineering handbook. (CRC press, 2018).
24. Li, W., Azam, S., Dai, G. & Fan, Z. Prussian blue based vertical graphene 3D structures for high frequency electrochemical capacitors. *Energy Storage Materials* **32**, 30–36 (2020).
25. Jones, M. Valve amplifiers. (Elsevier, 2011).
26. Gamby, J., Taberna, P., Simon, P., Fauvarque, J. & Chesneau, M. Studies and characterisations of various activated carbons used for carbon/carbon supercapacitors. *J. power sources* **101**, 109–116 (2001).
27. Yuan, C., Zhang, X., Wu, Q. & Gao, B. Effect of temperature on the hybrid supercapacitor based on NiO and activated carbon with alkaline polymer gel electrolyte. *Solid State Ionics* **177**, 1237–1242 (2006).
28. Qiu, J. *et al.* On-Chip MXene microsupercapacitors for AC-line filtering applications. *Adv. Energy Mater.* **9**, 1901061 (2019).
29. Miller, J. R. & Outlaw, R. A. Vertically-oriented graphene electric double layer capacitor designs. *J. Electrochem. Soc.* **162**, A5077–A5082 (2015).
