# Supplementary material for: Aqueous hybrid electrochemical capacitors with ultra-high energy density approaching for thousand-volts alternating current line filtering
Source: Nat Commun. 2022 Oct 26;13:6359. doi: 10.1038/s41467-022-34082-2 (PMC9606111; doi:10.1038/s41467-022-34082-2)
Supplement: Supplementary file 2 — Description of additional Supplementary File [file 41467_2022_34082_MOESM2_ESM.pdf]

**Descriptions of additional supplementary data files**

Supplementary Movie 1 Wetting state of CPN film in water

Supplementary Movie 2: Wetting state of p-CNT film in water
